# Chemical Constituents and Antiproliferative Activity Against RAFLs and HepG2 Cells of *Clematis henryi*

**DOI:** 10.3390/ijms262211216

**Published:** 2025-11-20

**Authors:** Bin Wang, Meng-Yun Wang, Yu-Pei Yang, Wei Su, Xin Jiang, Shi-Qi Liu, Qu-Jing Luo, Wen-Chao Zhou, Ling Liang, Hao Zheng, Qing-Ling Xie, Huang-He Yu, Yu-Qing Jian, Xu-Dong Zhou, Bin Li, Cai-Yun Peng, Wei Wang

**Affiliations:** TCM and Ethnomedicine Innovation & Development International Laboratory, School of Pharmacy, Hunan University of Chinese Medicine, Changsha 410208, China

**Keywords:** *Clematis henryi*, chemical composition, proliferation inhibition activity, RAFLs cells, HepG2 cells

## Abstract

*Clematis henryi* (*C. henryi*) is a valuable medicinal plant in the Tujia ethnic family, which is widely used for the treatment of rheumatism arthritis and limb numbness. There are few studies on the chemical composition and biological activity of *C. henryi* at present. In this study, we isolated and purified thirty-one compounds in total, including four new compounds (**1**, **29**–**31**) and twenty-seven known compounds (**2**–**28**). These isolated compounds were identified by a variety of spectroscopic data including NMR spectrometry and mass spectroscopy. These thirty-one compounds were tested for their proliferation inhibition activity on RAFLs and HepG2 cells. The results indicated that compound **29** and **30** exhibited weak inhibition of proliferation activity against RAFLs cells. Meanwhile, compounds **8**, **10**, **29**, and **30** exhibited moderate inhibition of proliferation activity on HepG2 cells with an IC_50_ value between 16.07 and 19.83 µM. The results of this study could serve as a reference for the further comprehensive utilization of *C. henryi*.

## 1. Introduction

**Figure 1:**
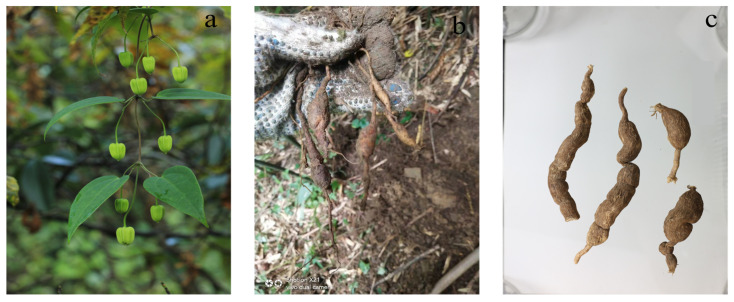
Various plant organs of *C. henryi*. (**a**) Aerial parts and flower buds; (**b**) freshly collected roots; (**c**) root specimens.

*C*. *Henryi* (Figure 1) is a widely used medicine plant in the Tujia family [1], locally named “Dilei” or “Xuelikai” [2], which is mainly distributed in southern China and generally grows in ravines at an altitude of 500–1400 m [3,4]. *C. henryi* has the effect of circulating blood, dispersing blood stasis, regulating qi-flowing, alleviating pain, and is often used for various therapeutic purposes such as healing bruises, stomach and abdominal pain, bronchitis, and mumps [5,6]. Pharmacological studies have shown that *C. henryi* has obvious sedative and analgesic effects on head, stomach, abdominal muscle, and joint pain [7], and also has antibacterial, antitumor, analgesic, and diuretic effects [3,6,8]. *Clematis* plants have been discovered to have a wide range of activities. Literature reports indicate that the anti-RA effects of *Clematis* plants primarily rely on pathways such as anti-inflammation and analgesia. The underlying mechanisms may involve inhibiting the expression of inflammatory cytokine like TNF-α, IL-6, IL-1β, PGE2, and COX-2 [9,10,11,12,13] and suppressing synovial cell hyperplasia [14,15,16,17] to reduce serum inflammation levels, alleviating the inflammatory response and clinical symptoms. Research by Sun [18] et al. further confirmed that total saponins from *C. henryi* significantly alleviated hind paw swelling in collagen-induced arthritis model rats, reduced serum levels of inflammatory markers such as IgG, IL-1β, and TNF-α, and markedly inhibited synovial hyperplasia, demonstrating anti-arthritis potential.

**Figure 2:**
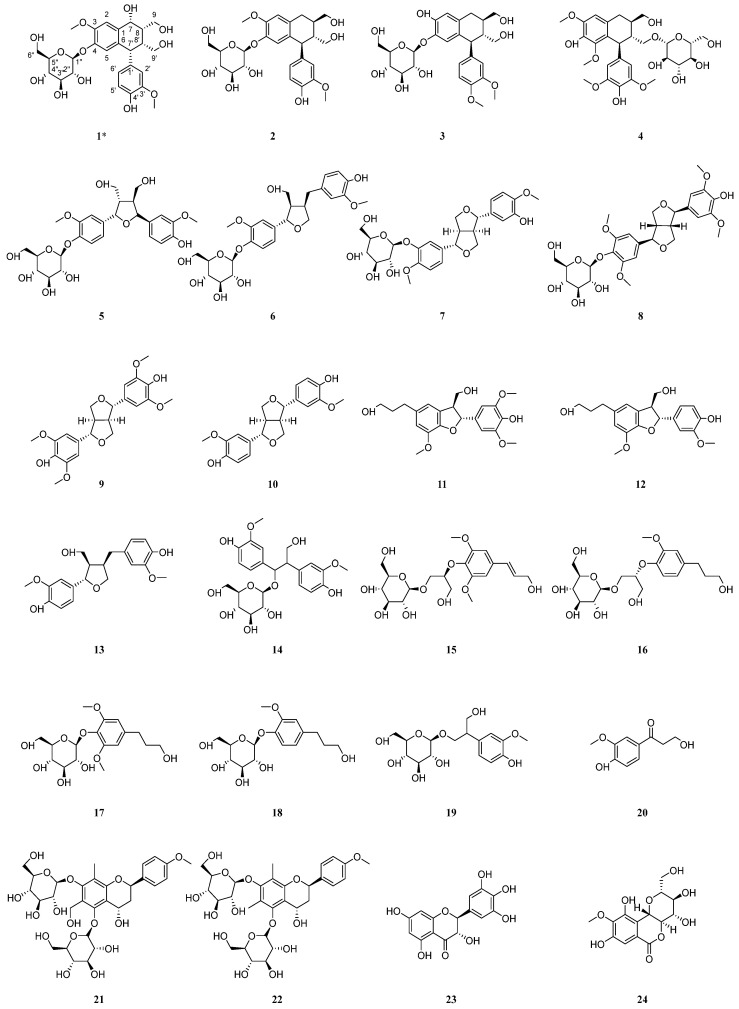

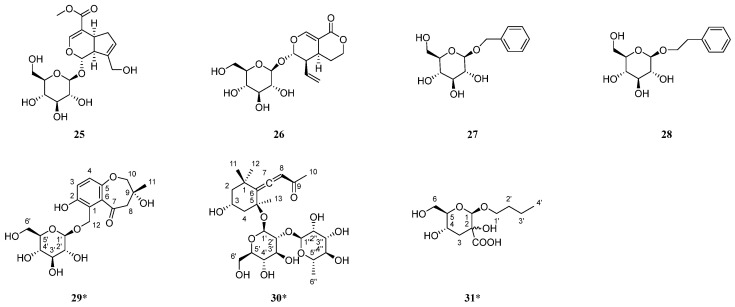
Structures of isolated compounds from the roots of *C. henryi.* * means new compound.

Currently, *C. henryi* receives more attention due to its good anti-arthritis effect [19]. Meanwhile, there is little literature reporting on its chemical composition [5,6,20]. Due to our efforts to isolate natural products, thirty-one compounds were isolated from the alcoholic extract of *C. henryi* roots in the present work, including four new compounds and twenty-seven known compounds (Figure 2). Furthermore, the anti-cell proliferation activity on RAFLs and HepG2 cells of all the isolated compounds were investigated. The results indicated that compound **29** and **30** exhibited weak activity against RAFLs cells and compounds **8**, **10**, **29**, and **30** exhibited moderate inhibition of proliferation activity on HepG2 cells with an IC_50_ value between 16.07 and 19.83 µM. Meanwhile, we presented the details of the isolation and structure identification of the isolated compounds.

## 2. Results

### 2.1. Structure Elucidation

**Figure 3:**
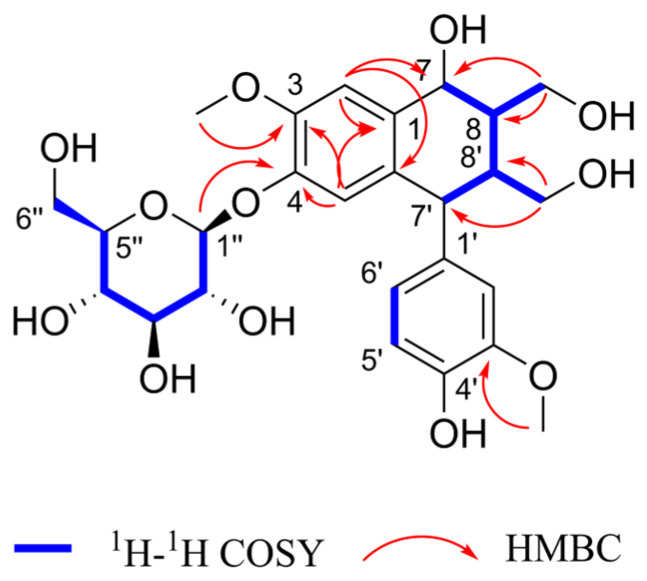
Key HMBC and ^1^H-^1^H COSY correlations of compound **1**.

Henriside A (compound **1**) was obtained as a light gray powder. The molecular formula C_26_H_34_O_12_ was indicated by the [M + Na]^+^ peak at *m*/*z* 561.1942 (calcd for C_26_H_34_O_12_Na^+^, 561.1943) in the HR-ESI-MS and supported by the ^13^C-NMR data (Table 1). Compound **1** with 10 degrees of unsaturation been presumed to contain two benzene rings. The ^1^H-NMR (Figure S1) spectrum of compound **1** showed five aromatic proton signals [*δ*_H_ 6.99 (1H, s), 6.92 (1H, s), 6.78 (1H, d), 6.71 (1H, d), and 6.56 (1H, dd)] with a set of ABX coupling proton signals of benzene ring [*δ*_H_ 6.78 (1H, d, *J* = 2.1 Hz, H-2′), 6.71 (1H, d, *J* = 8.2 Hz, H-5′), and 6.56 (1H, dd, *J* = 8.2, 2.1 Hz, H-6′)]. Four hypomethyl protons [4.85 (1H, m), 4.14 (1H, s), 2.87 (1H, s), 2.45 (1H, s)] which hypomethyl proton [4.85 (1H, m)] were connected with C-7 (*δ*_C_79.8). Two sets of hydroxymethyl proton singlets [*δ*_H_ 3.42 (1H, m), 3.20 (1H, dd), 4.16 (1H, dd), 3.82 (1H, d)] were attributed to H-9 and H-9′. Two groups of methoxy signals [*δ*_H_ 3.91 (3H, s), 3.79 (3H, s)] are linked at C-3 and C-3′ from the HMBC correlation of H-3-OCH_3_ and H-3′-OCH_3_, respectively, with C-3 (*δ*_C_56.8) and C-3′ (*δ*_C_56.5). The ^13^C NMR and DEPT spectrum (Figures S2 and S3) of compound **1** exhibited 20 carbon resonances corresponding to the above portions. The carbon signal [*δ*_C_ 103.0, 78.2, 77.8, 74.8, 71.2, 62.3] and the corresponding hydrogen signal suggest the presence of glucose. These spectral data indicate that compound **1** has a similar structure with the compound cycloolivil 6-*O*-*β*-*D*-glucopyranoside [21,22], and the difference in substituent location between them is the hydroxyl group shifting from C-8 to C-7. Meanwhile, the glucose group linked at C-4 by the proof of HMBC cross-peak of H-1” was correlated with its C-4 (*δ*_C_147.6). The detailed analysis of the 2D NMR spectra (Figures S4–S6) are shown in Figure 3. The stereochemistry of compound **1** was determined by the CD and NOESY spectra. Through comprehensive analysis and calculation, the absolute configuration at C-7, C-8, C-7′, and C-8′ could be 7*R*, 8*S*, 7′*S*, and 8′*R*. In a word, the structure of compound **1** was established as shown below.

**Figure 4:**
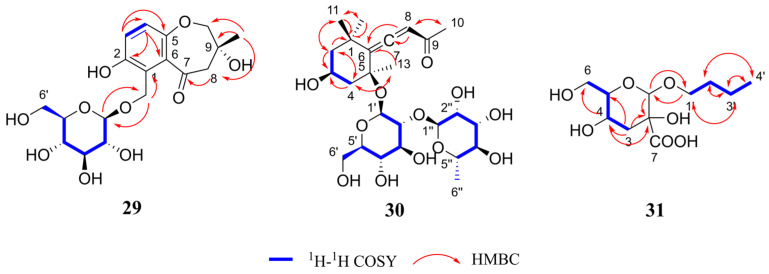
Key HMBC and ^1^H-^1^H COSY correlations of compound **29**–**31**.

Henriside B (compound **29**) was obtained as a light gray powder. The molecular formula C_18_H_24_O_10_ was indicated by the [M + Na]^+^ peak at *m*/*z* 423.1259 (calcd for C_18_H_24_O_10_Na^+^, 423.1262) in the HR-ESI-MS and supported by the ^13^C-NMR data (Table 2). Detailed analysis for 1D and 2D NMR data (Figures S7–S12) indicated that compound **29** inferred that there may be a tetrasubstituted benzene ring structure by proton signals [*δ*_H_ 6.92 (1H, d, *J* = 8.8 Hz, H-4), 6.86 (1H, d, *J* = 8.8 Hz, H-3)] and carbon signal [*δ*_C_ 123.2 (C-1), 153.2 (C-2), 120.3 (C-3), 122.5 (C-4),154.7 (C-5), 132.9 (C-6)]. In addition, the presence of a glucose moiety was evidenced by the characteristic signals (*δ*_C_ 103.7 (C-1′), 75.1 (C-2′), 78.0 (C-3′), 71.5 (C-4′), 78.1 (C-5′), 62.7 (C-6′)) along with the ^1^H-^1^H COSY and HMBC spectra. Moreover, the cross-peak in the HMBC spectra between *δ*_H_ 4.31 (1H, d, *J* = 7.9 Hz, H-1′) and *δ*_C_ 63.0 (C-12) demonstrated the glucose group was attached at C-12. The NMR data revealed that compound **29** is structurally analogous to hexapetoside C [23], featuring saturation of the C-8,9 double bond, an additional hydroxyl group at C-9, and the concurrent presence of a carbonyl group at C-7. In summary, the structure of compound **29** was determined as shown Figure 2 and the key HMBC and ^1^H-^1^H COSY correlations are shown in Figure 4.

Henriside C (compound **30**) was obtained as a light gray gel. The molecular formula C_25_H_40_O_12_ was indicated by the [M + Na]^+^ peak at *m*/*z* 555.2408 (calcd for C_25_H_40_O_12_Na^+^, 555.2412) in the HR-ESI-MS and is supported by the ^13^C-NMR data (Table 2). The comparison of ^1^H and ^13^C NMR data (Figures S13–S18) revealed that the compound **30** is structurally identical to citroside A [24,25], but bears an additional rhamnose unit (*δ*_C_ 101.6 (C-1″), 72.2 (C-2″), 72.3 (C-3″), 74.1 (C-4″), 69.8 (C-5″), 18.1 (C-6″)). The structure of compound **30** was determined as shown Figure 2 and the key HMBC and ^1^H-^1^H COSY correlations are shown in Figure 4.

Henriside D (compound **31**) was obtained as colorless oil material. The molecular formula C_11_H_20_O_7_ was indicated by the [M + H]^+^ peak at *m*/*z* 265.1295 (calcd for C_11_H_21_O_7_^+^, 265.1282) in the HR-ESI-MS and is supported by the ^13^C-NMR data (Table 2). The ^13^C NMR spectrum revealed the presence of a carbonyl group in the compound, which was further determined to be connected to a quaternary carbon at the C-2 position through HMBC correlations. The NMR spectra (Figures S19–S24) revealed that the compound **31** is similar to butyl-α-D-glucopyranoside [26]. However, the altered chemical shift at C-3, coupled with the presence of two hydrogen atoms at this position, indicated the absence of a hydroxyl substitution at C-3 compared with butyl-α-D-glucopyranoside. The structure of compound **31** is shown Figure 2 and the key HMBC and ^1^H-^1^H COSY correlations are shown in Figure 4.

In addition, the structures of the twenty-seven known compounds were identified as asisolariciresinol-4-*O*-*β*-D-glucopyranoside (**2**) [27], sargentodosideB (**3**) [28], (+)-lyonirenisol-3*α*-*O*-*β*-D-glucopyranoside (**4**) [29], neoolivil-4-*O*-*β*-D-glucoside (**5**) [30], lariciresinol-4-*O*-*β*-D-glucopyranoside (**6**) [31], 4,4′-dimethoxy-3′-hydroxy-7,9′:7′,9-diepoxylignan-3-*O*-*β*-D-glucopyranoside (**7**) [32], (+)-syringaresinol-4-*O*-*β*-D-glucopyranoside (**8**) [33], syringaresinol (**9**) [33], pinoresinol (**10**) [34], (+)-7*R*,8*S*-5-methoxydihydrodehydroconiferyl alcohol (**11**) [35], (7*S*,8*R*)-didydrodehydrodiconiferyl alcohol (**12**) [36], lariciresinol (**13**) [37], hovetrichosideA (**14**) [38], (−)-(2*R*-)-1-*O*-*β*-D-glucopyranosyl-2-{2,6-dimethoxy-4-[1-(*E*)-propen-3-ol]phenoxyl}propane-3-ol (**15**) [39], (−)-(2*R*)-1-*O*-(*β*-d-glucopyranosyl)-2-[2-methoxy-4-(ω-hydroxypropyl)-phenoxyl]-propan-3-ol (**16**) [40], dihydrosyringin (**17**) [41], dihydroconiferin (**18**) [40], junipediolA-8-*O*-*β*-D-glucoside (**19**) [42], ω-hydroxypropioguaiacone (**20**) [43], triphyllinA (**21**) [44], eruberinB (**22**) [45], dihydromyricetin (**23**) [46], bergenin (**24**) [47], geniposide (**25**) [48], sweroside (**26**) [49], Benzyl-*β*-D-glucopyranoside (**27**) [50], and 2-phenylethyl-*β*-D-glucopyranoside (**28**) [51] by comparing their spectroscopic data with those reported in the literature.

### 2.2. Anti-Proliferation Activity

#### 2.2.1. Anti-Proliferative Activity on HFLs Cell 

**Figure 5:**
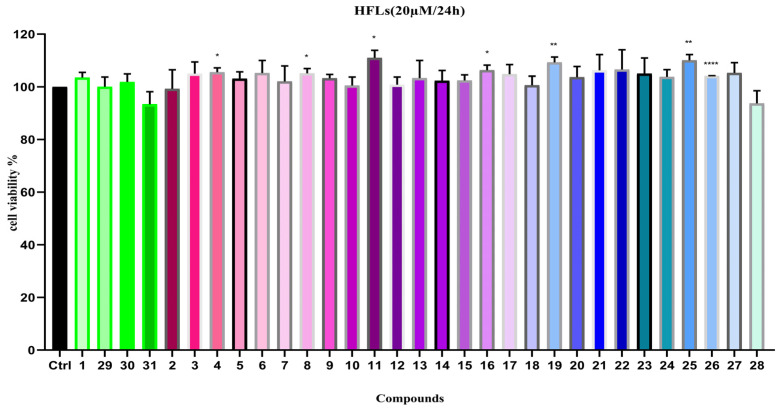
Cell viability of compounds **1**–**31** on HFLs (20 µM). Note: n = 3, mean ± SD. Compared with the control: **** *p* < 0.0001, ** *p* < 0.01 and * *p* < 0.05.

To evaluate the safety profile of the compounds (1–31) on HFLs cells, a 24 h treatment at a concentration of 20 µM was conducted. The results demonstrated that, compared to the control group (cells only without drug intervention), including the new compound, **1** and **29**–**31** showed almost no inhibitory effects on HFLs cell growth (Figure 5). Furthermore, most compounds displayed a certain degree of growth-promoting effect, indicating that these compounds are non-cytotoxic to HFLs cell proliferation.

#### 2.2.2. Anti-Proliferative Activity on RAFLs Cell

**Figure 6:**
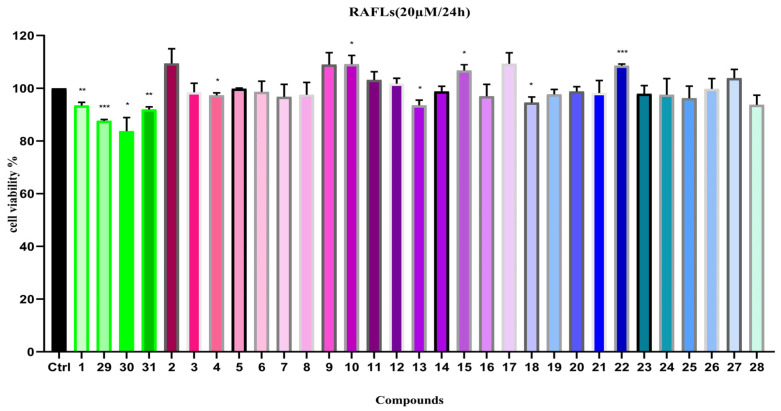
Cell viability of compounds **1**–**31** on RAFLs (20 µM). Note: n = 3, mean ± SD. Compared with the control: *** *p* < 0.001, ** *p* < 0.01 and * *p* < 0.05.

The anti-RA activity of compounds **1**–**31** was assessed by testing the proliferation rate of RAFLs cells in the experiment. The results revealed that, compared to the control group (cells inoculated without drug intervention), compounds **29** and **30** exhibited weak inhibitory activity on the proliferation of RAFLs cells (Figure 6), while the other compounds showed almost no inhibitory effect on the growth of RAFLs cells.

Moreover, in the experiments, we also compared the inhibitory effects of four new compounds, **1** and **29**–**31**, on RAFLs cell proliferation under a series of drug concentrations and treatment durations. The results demonstrated that, when the exposure time of the new compounds, **1** and **29**–**31**, to RAFLs cells was extended from 24 h to 48 h, it did not show a notable inhibitory effect on the proliferation of RAFLs cells.

#### 2.2.3. Anti-Proliferative Activity on HepG2 Cell

**Figure 7:**
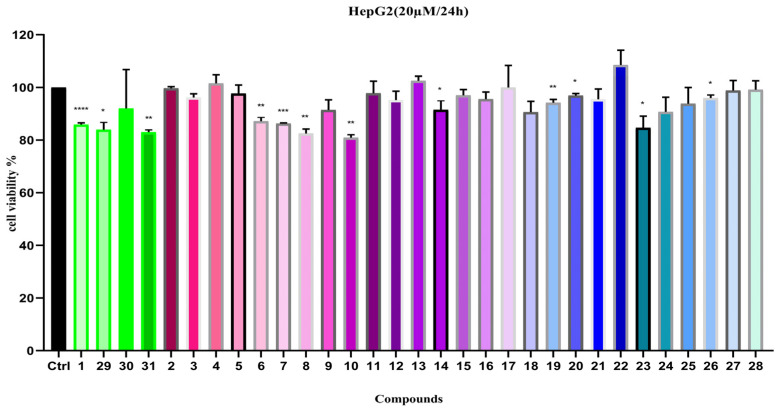
Cell viability of compounds **1**–**31** on HepG2 (20 µM). Note: n = 3, mean ± SD. Compared with the control: **** *p* < 0.0001, *** *p* < 0.001, ** *p* < 0.01 and * *p* < 0.05.

Since a number of lignan compounds were isolated and identified from *C. henryi*. in this study, we evaluated their hepato-protective activity by testing the inhibitory effects of these compounds on HepG2 cell proliferation at a concentration of 20 µM (Figure 7). The results show that compounds **1**, **8**, **10**, **29**, and **31** demonstrated moderate inhibitory activity on HepG2 cell proliferation, while the remaining compounds had no significant effects.

Furthermore, we use paclitaxel (PTX 0.5 µM) as the positive control to investigate the effects of compounds **1**, **8**, **10**, and **29**–**31** on HepG2 cell growth at different concentrations and treatment periods. The experimental results indicated that, when the treatment duration was extended from 24 h to 48 h, the inhibitory effects of compounds **8**, **10**, and **30** on HepG2 cell proliferation increased, with IC_50_ values ranging between 17 and 20 µM (Table 3).

## 3. Discussion

This study selected *C. henryi* as the research subject for two primary reasons: firstly, this plant has had a folk use for treating arthritis for a long time; secondly, plants from the *Clematis* genus are generally recognized for their efficacy in “expelling wind and unblocking collaterals” and are commonly used to treat rheumatic arthralgia. Especially, plants such as *C. chinensis* and *C. armandii* have been officially documented in the Chinese pharmacopeia, further validating the medicinal value of this genus in the field of rheumatism treatment.

The literature reports that lignan compounds possess a wide range of activities, including anti-inflammatory [34,38,52,53,54,55,56], antitumor [28,33,57,58,59], hepatoprotective [60,61,62,63], and antioxidant [64,65,66] effects. For instance, lignans such as (+)-syringaresinol, (+)-pinoresinol, herpetol [34], simulanol, 5′-methoxylariciresinol, nectandrin B [55], bruceine L, and cleomiscosin A [56] have been confirmed to exert anti-inflammatory effects by inhibiting NO production in LPS-induced RAW 264.7 cells. Loniceralanside A [52] exerts anti-inflammatory effects by inhibiting PAF-induced β-glucuronidase release in rat polymorphonuclear neutrophils. Sesamol [53] exhibits anti-inflammatory properties by inhibiting the secretion of various pro-inflammatory factors like IL-1β and TNF-α, and downregulating key signaling pathways such as NF-κB and MAPK. Houpulin G/I/J [54] exerts anti-inflammatory activity by inhibiting Fmlp/CB-induced superoxide anion generation and elastase release in human neutrophils. Additionally, Sargentol and Cinchonains Ia inhibit the proliferation of Hela and Siha cells [28]. Hanultarin exhibits moderate cytotoxic activity against A549, SK-OV-3, SK-MEL-2, and HCT15 cell lines [33]. Schisandrin B can induce pyroptosis in HepG2 cells by activating NK cell-mediated antitumor immunity [57]. Total lignans from *Syringa pinnatifolia* and *Schisandra chinensis* have also been confirmed to possess anti-hepatoma activity [58,59]. These studies provide strong support for the anti-inflammatory and antitumor activities of lignan compounds.

In this study, 31 compounds were isolated and identified from *C. henryi*, predominantly lignans, including 4 new compounds. Given the limited number and structural diversity of the new compounds, further systematic chemical constituent research is necessary to obtain more structurally similar compounds for in-depth analysis of their structure–activity relationships. The literature indicates that the characteristic chemical constituents of the *Clematis* genus are primarily saponins, additionally containing lignans and flavonoids. However, this study mainly obtained lignans, showing a significant difference from the characteristic chemical profile in this genus, such as *C. chinensis* Osbeck, which is dominated by triterpenoid saponins. Therefore, more efforts should focus on the targeted isolation and activity research of saponins of *C. henryi*.

Based on traditional usage and the isolated compounds, this study evaluated the inhibitory effects of compounds **1**–**31** on the proliferation of RAFLs and HepG2 cells. The experiment first assessed the safety of **1**–**31** on normal FHLs cells. Results showed that, at a concentration of 20 µM, most compounds did not inhibit the proliferation of normal FHLs cells. In the RAFLs model, only compounds **29** and **30** exhibited weak inhibitory activity (Figure 6). The four new compounds (**1**, **29**–**31**) did not demonstrate significant proliferation inhibitory effects at different concentrations and treatment durations (extended from 24 to 48 h). In the HepG2 cell proliferation inhibition assay, compounds **1**, **8**, **10**, **29**, and **31** exhibited moderate inhibitory activity at 20 µM. Further experiments using paclitaxel (PTX, 0.5 µM) as a positive control revealed that, extending the treatment time to 48 h, the inhibitory activities of compounds **8**, **10**, and **30** were little enhanced, with IC_50_ values ranging between 17 and 20 µM (Table 3), indicating a certain degree of time-dependent inhibition. For compound **29**, the increase in IC_50_ with longer exposure durations also indicates a time-dependent pharmacological effect. We think these time-dependent IC_50_ values warrant further attention and could be explored in future studies.

The majority of the 31 compounds were lignans or phenylpropanoids, none of which exhibited activity against RAFLs cells or safety to HFLs cells, and two structurally distinct compounds (**29** and **30**) demonstrated only minimal inhibitory effects. Compounds **8** and **10**, both lignans, exhibited moderate inhibitory activity against HepG2 cell proliferation. By comparing the structures of compounds **7**–**10**, we hypothesize that the sugar moiety and methoxy groups may influence the activity, though this requires further data validation. With the exception of **29** and **30**, none of the other compounds demonstrated significant effects on HepG2 cell proliferation. This indicates that this class of compounds does not represent the active constituents of the *Clematis* species. However, both **29** and **30** exhibited moderate activity against the RAFLs and HepG2 cell lines. While these compounds share common structural features—a carbonyl group and a sugar moiety—their overall structures differ significantly. Therefore, whether the carbonyl group and sugar moiety serve as the key pharmacophores requires further investigation.

In summary, among the 31 compounds isolated from *C. henryi* in this study, only a few (**8**, **10**, **29**, and **30**) exhibited relatively weak inhibitory activity against HepG2 cell proliferation, while most compounds showed almost no inhibitory effects on the proliferation of either RAFLs or HepG2 cells. Considering traditional application experience and literature reports, we speculate that the therapeutic effects of *C. henryi* may result from the synergistic effects of multiple types of compounds, or that the key active components inhibiting RAFLs proliferation have not yet been isolated in this study, or the characteristic compounds with significant bioactivity are present in low abundance and were not isolated. Therefore, future research should focus on some aspects: systematically isolating chemical constituents, strengthening the targeted isolation and activity research on saponins, establishing more relevant activity evaluation models that better reflect the RA pathological process, such as anti-inflammatory and immunomodulatory assays, etc. These efforts will provide more data for elucidating the effective material and action mechanism of *C. henryi*.

## 4. Materials and Methods

### 4.1. General Experimental Procedures

Optical rotations were measured with a Rudolph Research AutoPol IV (Rudolph Research Analytical, Hackettstown, NJ, USA). Circular dichroism (CD) spectra were recorded on a JASCO J-1500-150 spectropolarimeter (JASCO Corporation, Tokyo, Japan). UV spectra (methanol) were measured with a Hewlett-Packard 8452AUV-vis spectrophotometer (Hewlett Packard Enterprise, Spring, TX, USA). One-dimensional and two-dimensional NMR spectra were recorded on a Bruker AV-600 NMR spectrometer with TMS as internal standard and all chemical shifts (*δ*) are reported in ppm relative to the solvent signals, and coupling constants are reported in Hz (Bruker Corporation, Bremen, Germany). HR-ESI-MS data were acquired on an Agilent infinity 1290 mass spectrometer (Agilent Technologies, Santa Clara, CA, USA). Semi-preparative HPLC was conducted on an Agilent 1260 Infinity II HPLC system with an Eclipse XDB-C18 column (250 mm × 4.6 mm, 5 μm) (Agilent Technologies, Santa Clara, CA, USA). Column chromatography (CC) was performed with silica gel (100–200 and 200–300 mesh) (Qingdao Marine Chemical Factory, Qingdao, China), ODS RP-C_18_ gel (Sigma-Aldrich, St. Louis, MO, USA), Sephadex LH-20 (Shanghai Aladdin Biochemical Technology Co., Ltd. Shanghai, China), and Macroporous adsorbent resin AB-8 (Shanghai Yuanye Bio-Technology Co., Ltd. Shanghai, China). Thin layer chromatography (TLC) was employed to monitor the CC fractions, with visualization achieved through the application of 1% vanillin in H_2_SO_4_ as a spraying reagent.

### 4.2. Chemicals and Reagents

HFLs cells, RAFLs cells, and HepG2 cells (Shanghai Fuheng Biotechnology Co., Ltd. Shanghai, China), CCK-8 (Beijing Solarbio Science & Technology Co., Ltd. Beijing, China), Methotrexate and Paclitaxel (Beijing Danda Biological Technology Co., Ltd. Beijing, China), DMEM high glucose medium, MEM medium, 0.25% trypsin solution (Wuhan Procell Life Science & Technology Co., Ltd. Wuhan, China), DMSO and Na_2_CO_3_ Solution (Shanghai Macklin Biochemical Technology Co., Ltd. Shanghai, China), PBS Buffer (Cytiva, Marlborough, MA, USA), methanol and acetonitrile (HPLC grade, Sigma-Aldrich (Wuxi) Life Science & Tech. Co., Ltd. Wuxi, China), anhydrous ethanol, dimethyl sulfoxide (DMSO), and other analytical grade chemical (Sinopharm Chemical Reagent Co., Ltd. Shanghai, China), purified water (C’estbon Beverage (China) Co., Ltd. Shenzhen, China).

### 4.3. Plant Material

The roots of *C. henryi* were collected in October 2018 from Huping Mountain in Shimen County, Changde, Hunan, China. They were identified by Professor Wang Wei from Hunan University of Chinese Medicine (Changsha, China). The samples were stored in the TCM and Ethnomedicine Innovation & Development International Laboratory, Hunan University of Chinese Medicine, Changsha, Hunan, China.

### 4.4. Extraction and Isolation

The extract (367 g, 8.2% yield) from roots of *C. henryi* (4.5 kg) was obtained by macerating with 70% ethanol (25 L) at room temperature three times. The extract was concentrated under reduced pressure at a water bath temperature of 45 °C to obtain the total extract (367 g). The total extract was suspended in water and successively partitioned with petroleum ether (PE), ethyl acetate (EtOAc), and n-butanol (n-BuOH), yielding the PE fraction (26.7 g), EtOAc fraction (26.2 g), n-butanol fraction (80.1 g), and the aqueous fraction (220.0 g).

The EtOAc fraction (26.2 g) was separated by silica gel column chromatography and eluted with a DCM-MeOH gradient system (50:1 to 0:1, *v*/*v*) to yield 11 fractions (Fr.1–Fr.11). Fr.4 (4.3 g) was subjected to silica gel column chromatography, yielding 10 subfractions (Fr.4.1–Fr.4.10). Subfraction Fr.4.8 (200.0 mg) was separated by silica gel column chromatography to give four subfractions (Fr.4.8.1–Fr.4.8.4). Fr.4.8.4 was then purified by semi-preparative HPLC (40%MeOH; 2.0 mL/min; 210 and 254 nm) to afford compound **20** (16.5 min, 1.9 mg). Fr.8 (2.0 g) was separated by silica gel column chromatography, yielding nine subfractions (Fr.8.1–Fr.8.9). Subfraction Fr.8.6 (529.9 mg) was eluted by silica gel column chromatography to give five subfractions (Fr.8.6.1–Fr.8.6.5). Fr.8.6.3 (296.3 mg) was then subjected to ODS column chromatography, yielding 26 subfractions (Fr.8.6.3.1–Fr.8.6.3.26). Fr.8.6.3.12 (13.1 mg) was purified by semi-preparative HPLC (8%ACN; 3.0 mL/min; 210 and 254 nm) to afford compound **7** (45.1 min, 4.8 mg). Fr.8.6.3.17 (39.7 mg) was separated by semi-preparative HPLC (31%MeOH; 3.0 mL/min; 210 and 254 nm) to yield compound **8** (42.4 min, 1.9 mg). Fr.8.6.3.26 (15.1 mg) was separated by semi-preparative HPLC (15%ACN; 3.0 mL/min; 210 and 254 nm) to yield compounds **27** (16.3 min, 0.9 mg) and **28** (31.3 min, 0.8 mg). Finally, Fr.8.6.4 (102.9 mg) was purified by semi-preparative HPLC (15%ACN; 2.0 mL/min; 210 and 254 nm) to afford compound **24** (15.7 min, 4.5 mg).

The *n*-BuOH fraction (80.1 g) was preliminarily separated using a macroporous resin adsorption column and eluted with an H_2_O-EtOH gradient system (1:0 to 0:1, *v*/*v*) to yield four fractions (Fr.1–Fr.4). Fr.2 (10.9 g) was subjected to ODS column chromatography, yielding 12 subfractions (Fr.2.1–Fr.2.12). Fr.2.2 (3.3 g) was separated by silica gel column chromatography to afford 11 subfractions (Fr.2.2.1–Fr.2.2.11). Fr.2.2.7 (152.1 mg) was eluted by ODS column chromatography to afford compound **31** (2.7 mg). Fr.2.2.8 (111.8 mg) was purified by semi-preparative HPLC (4%ACN; 2.0 mL/min; 210 and 254 nm) to yield compound **19** (48.0 min, 7.0 mg). Fr.2.5 (2.7 g) was separated by silica gel column chromatography, yielding nine subfractions (Fr.2.5.1–Fr.2.5.9). Fr.2.5.2 (106.9 mg) was subjected to gel column chromatography to afford 10 subfractions (Fr.2.5.2.1–Fr.2.5.2.10). Fr.2.5.2.2 (48.9 mg) was purified by semi-preparative HPLC (30%MeOH; 2.0 mL/min; 210 and 254 nm) to yield compound **23** (34.1 min, 2.5 mg). Fr.2.5.5 (324.5 mg) was separated by gel column chromatography, yielding nine subfractions (Fr.2.5.5.1–Fr.2.5.5.9). Fr.2.5.5.3 (225.1 mg) was separated by semi-preparative HPLC (12%ACN; 2.0 mL/min; 210 and 254 nm) to afford compounds **18** (24.9 min, 1.5 mg) and **26** (2.2 min, 14.8 mg). Fr.2.5.5.3.2 (6.4 mg) was purified by semi-preparative HPLC (7%ACN; 2.0 mL/min; 210 and 254 nm) to yield compound **17** (77.2 min, 1.7 mg). Fr.2.5.6 (167.3 mg) was subjected to gel column chromatography, yielding seven subfractions (Fr.2.5.6.1–Fr.2.5.6.7). Fr.2.5.6.4 (64.8 mg) was separated by semi-preparative HPLC (11%ACN; 2.0 mL/min; 210 and 254 nm) to afford compound **25** (58.3 min, 14.5 mg). Fr.2.5.6.4.7 (22.3 mg) was purified by semi-preparative HPLC (9%ACN; 2.0 mL/min; 210 and 254 nm) to yield compounds **15** (159.5 min, 4.1 mg) and **3** (193.9 min, 6.3 mg). Fr.2.5.6.5 (31.5 mg) was separated by semi-preparative HPLC (9%ACN; 2.0 mL/min; 210 and 254 nm) to afford compounds **14** (54.3 min, 3.4 mg), **29** (63.2 min, 3.1 mg), **1** (71.6 min, 4.3 mg), and **2** (151.1 min, 7.4 mg). Fr.2.5.7 (319.3 mg) was subjected to gel column chromatography, yielding six subfractions (Fr.2.5.7.1–Fr.2.5.7.6). Fr.2.5.7.1 (35.7 mg) was purified by semi-preparative HPLC (13%ACN; 3.0 mL/min; 210 and 254 nm) to yield compound **30** (36.9 min, 1.8 mg). Fr.2.5.7.3 (73.8 mg) was separated by semi-preparative HPLC (8%ACN; 3.0 mL/min; 210 and 254 nm) to afford compound **16** (46.1 min, 1.0 mg). Fr.2.6 (298.7 mg) was separated by silica gel column chromatography, yielding 10 subfractions (Fr.2.6.1–Fr.2.6.10). Fr.2.6.7 (21.2 mg) was purified by semi-preparative HPLC (20% ACN; 2.0 mL/min; 210 and 254 nm) to afford compound **4** (14.1 min, 7.7 mg). Fr.2.12 (5.2 g) was separated by silica gel column chromatography, yielding six subfractions (Fr.2.12.1–Fr.2.12.6). Fr.2.12.1 (60.0 mg) was purified by semi-preparative HPLC (35%MeOH; 3.0 mL/min; 210 and 254 nm) to afford compounds **13** (39.5 min, 3.3 mg), **9** (63.2 min, 1.3 mg), and **10** (70.5 min, 1.2 mg). Fr.2.12.2 (62.4 mg) was separated by semi-preparative HPLC (18%ACN; 3.0 mL/min; 210 and 254 nm) to yield compounds **11** (58.3 min, 2.3 mg) and **12** (63.8 min, 3.8 mg). Fr.2.12.4 (41.7 mg) was purified by semi-preparative HPLC (17%ACN; 3.0 mL/min; 210 and 254 nm) to afford compound **5** (23.9 min, 0.7 mg). Fr.2.12.5 (331.6 mg) was subjected to gel column chromatography, yielding five subfractions (Fr.2.12.5.1–Fr.2.12.5.5). Fr.2.12.5.4 (39.7 mg) was separated by semi-preparative HPLC (20%ACN; 3.0 mL/min; 210 and 254 nm) to yield compound **6** (15.7 min, 1.9 mg). Fr.2.12.6 (331.6 mg) was eluted by ODS column chromatography, yielding 13 subfractions (Fr.2.12.6.1–Fr.2.12.6.13). Fr.2.12.6.8 (62.1 mg) was purified by semi-preparative HPLC (18%ACN; 3.0 mL/min; 210 and 254 nm) to afford compounds **21** (44.4 min, 6.1 mg) and **22** (52.0 min, 3.7 mg).

### 4.5. Anti-Proliferation Activity Assay

#### 4.5.1. Anti-HFLs Cell Proliferation Activity

The cells used in this study were obtained from a commercial company and were subsequently maintained and passaged in our laboratory. HFLs cells were maintained in DMEM F12 medium supplemented with 10% fetal bovine serum and 1% penicillin–streptomycin mixture. The cells were seeded in culture flasks and placed in a 5% CO_2_ incubator at 37 °C for routine cultivation. The proliferation inhibition activity on HFLs cells of the isolated samples was evaluated on cell viability by a CCK-8 assay. The experiment was set up with sample groups (concentration of 20 µM for all samples), a control group (cells only), and a blank group (culture medium only). Each group was set up with three replicate wells.

**Figure 8:**
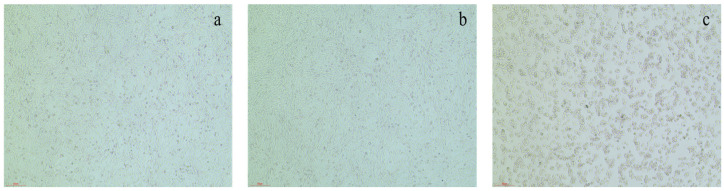
Cells in the logarithmic growth phase. (**a**) HFLs; (**b**) RAFLs; (**c**) HepG2.

The testing was conducted in 96-well plates, and 100 µL of HFLs cells (6 × 10^4^ cells/mL) in logarithmic growth phase (Figure 8) were seeded in each well and cultured for 24 h. After that, the culture plate was removed from the incubator, the medium was aspirated, and 100 µL of the sample solution (20 µM) was added to each well of the sample groups, while 100 µL of the corresponding culture medium was added to each well of the control and blank groups. The plate was subsequently returned to the 37 °C, 5% CO_2_ incubator for continued cultivation for 24 h. Then, the medium was aspirated again and 100 µL of the prepared CCK-8 dilution was added to each well, followed by continued incubation of 0.5 h. Subsequently, the optical density (OD) of each group was measured at 490 nm using a microplate reader. The cell survival rate was calculated based on the obtained data.

#### 4.5.2. Anti-RAFLs Cell Proliferation Activity

The preliminary assay procedure for evaluating the anti-proliferative effect of the samples on RAFLs cells is consistent with that described for HFLS cells. The experiment also tested the proliferation inhibitory activity of novel compounds **1** and **33**–**35** against RAFLs cells at various concentrations after 24 h and 48 h of treatment.

RAFLs cells were maintained in DMEM F12 medium supplemented with 10% fetal bovine serum and a 1% penicillin–streptomycin mixture. The cells (Figure 8) were seeded in culture flasks of 96-well plates and placed in a 5% CO_2_ incubator at 37 °C for routine cultivation. The inhibit proliferation activity on RAFLs cells of **1** and **33**–**35** were evaluated on cell viability by a CCK-8 assay. The experiment set up sample groups (concentrations of 10 µM, 20 µM, 30 µM, 40 µM, 50 µM, 60 µM, 80 µM, and 100 µM for compounds **1** and **33**–**35**), a positive control group (MTX 4.5 µM), a control group (cells only), and a blank group (culture medium only). Each group was set up with three replicate wells. A total of 100 µL of RAFLs cells (6 × 10^4^ cells/mL) in logarithmic growth phase were seeded in each well and cultured for 24 h. After that, 100 µL of **1** and **33**–**35** (10 µM, 20 µM, 30 µM, 40 µM, 50 µM, 60 µM, 80 µM, and 100 µM) was added to each well of the sample groups, while 100 µL of MTX and the corresponding culture medium was added to each well of the positive control and blank groups, respectively.

The plate was subsequently returned to the 37 °C, 5% CO_2_ incubator for continued cultivation for 24 and 48 h. Then, the medium was aspirated again and 100 µL of the prepared CCK-8 dilution was added to each well, followed by continued incubation for 0.5 h. Subsequently, the optical density (OD) of each group was measured at 490 nm using a microplate reader. The cell survival rate after 24 h and 48 h was calculated based on the obtained data.

The cell viability (%) of each test sample was calculated as follows:

Cell viability rate (%) = [(OD_sample_ − OD_blank_)/(OD_control_ − OD_blank_)] × 100%

### 4.6. Anti-HepG2 Cell Proliferation Activity

HepG2 cells were maintained in DMEM medium supplemented with 10% fetal bovine serum and 1% penicillin–streptomycin mixture. The cells (Figure 8) were seeded in culture flasks and placed in a 5% CO_2_ incubator at 37 °C for routine cultivation. The proliferation inhibitory activity of the samples was tested on cell viability by a CCK-8 assay. Sample groups (20 µM for all samples), the control group (cells only), and the blank group (culture medium only) were set up in the experiment. Each group was set up with three replicate wells. The experiment was conducted in 96-well plates, and 100 µL of HepG2 cells (6 × 10^4^ cells/mL) in logarithmic growth phase were seeded in each well and cultured for 24 h. After that, 100 µL isolated samples (20 µM) were added to each well of the sample groups, while 100 µL of the corresponding culture medium was added to each well of the control and blank groups. Removing the medium of plates after 24 h culturing, 100 µL of the prepared CCK-8 dilution was added to each well and incubated for 0.5 h, followed by measuring the absorbance at 490 nm using a microplate reader and calculating cell survival rate.

Repeating the above procedure, we also tested the proliferation inhibitory activity of compounds **1**, **8**, **10**, and **33**–**35** against HepG2 cells at various concentrations after 24 h and 48 h of treatment, and calculated the cell survival rate after 24 h and 48 h.

The cell viability (%) of each test sample was calculated as follows:

Cell viability rate (%) = [(OD_sample_ − OD_blank_)/(OD_control_ − OD_blank_)] × 100%

The IC_50_ values were calculated by using GraphPad Prism 8.0.

## 5. Conclusions

In our study, a comprehensive phytochemical investigation of *C. henryi* was conducted to isolate 31 compounds, including 27 known compounds and 4 new compounds. The structures of these compounds were elucidated through various chromatographic and spectroscopic methods. Furthermore, based on the traditional applications of *C. henryi* and the types of compounds isolated, we evaluated their proliferation inhibition activity against RAFLs and HepG2 cells. The results show that compounds **29** and **30** exhibited weak activity against RAFLs cells and compounds **8**, **10**, **29**, and **30** exhibited moderate inhibition of proliferation activity on HepG2 cells with an IC_50_ value between 16.07 and 19.83 µM. No highly active compounds were identified in these assays. It is speculated that the compounds isolated in this study may not be characteristic compounds of *C. henryi*.

*C. henryi* is traditionally used for treating RA in the Wuling Mountain region. The findings of this study enhance the understanding of *C. henryi* and provide valuable guidance for its further research. We need to isolate a greater diversity of compounds, especially the saponin compounds from *C. henryi*, to elucidate its bioactivity. Moreover, we also can aim to obtain more lignan compounds and try to explore their anti-hepatocarcinoma and hepatoprotective activity in the future.

## Figures and Tables

**Table 1:** The ^1^H NMR (600 MHz) and ^13^C NMR (151 MHz) data of the compound **1** (in CD_3_OD) (*δ* in ppm, J in Hz).

No.	*δ*_C_	*δ*_H_
1	129.3	-
2	120.8	6.99, s
3	149.8	-
3-OCH3	56.8	3.91, s
4	147.6	-
5	114.2	6.92, s
6	134.3	-
7	79.8	4.85, m
8	48.2	2.45, s
9	61.4	3.42, m 3.20, dd (5.6, 3.7)
1′	136.9	-
2′	113.9	6.78, d (2.1)
3′	148.7	-
3′-OCH3	56.5	3.79, s
4′	145.9	-
5′	115.7	6.71, d (8.2)
6′	122.5	6.56, dd (8.2, 2.1)
7′	50.8	4.14, s
8′	45.9	2.87, s
9′	76.2	4.16, dd (8.1, 5.2) 3.82, d (7.6)
1″	103.0	4.78, d (7.7)
2″	74.8	3.47, dd (9.0, 7.8)
3″	77.8	3.39, d (8.7)
4″	71.2	3.36, t (8.8)
5″	78.2	3.20, dd (5.6, 3.7)
6″	62.3	3.73, dd, (12.2, 2.2) 3.61, dd (12.1, 5.4)

**Table 2:** The ^1^H NMR (600 MHz) and ^13^C NMR (151 MHz) data of the **29**–**31** (in CD_3_OD) (*δ* in ppm, J in Hz).

No.	29		No.	30	
	*δ*_C_	*δ*_H_		*δ*_C_	*δ*_H_
1	123.2	-	1	37.0	-
2	153.2	-	2	49.8	1.92, ddd (12.5, 4.3, 2.0) 1.34, d (11.9)
3	120.3	6.86, d (8.8)	3	63.8	4.23, tt (11.3, 4.1)
4	122.5	6.92, d (8.8)	4	50.4	2.46, ddd (13.2, 4.3, 2.0) 1.42, m
5	154.7	-	5	78.1	-
6	132.9	-	6	118.6	-
7	203.4	-	7	201.0	-
8	58.0	3.11, d (11.4) 2.92, d (11.2)	8	101.4	5.93, s
9	74.1	-	9	213.0	-
10	82.6	4.00, d (12.4) 3.83, d (8.9)	10	26.7	2.19, s
11	25.1	1.28, s	11	30.4	1.38, s
12	63.0	5.10, d (11.1) 4.84, d (11.1)	12	32.5	1.16, s
1′	103.7	4.31, d (7.9)	13	25.6	1.49, s
2′	75.1	3.14, d (7.9)	1′	97.9	4.61, d (7.6)
3′	78.0	3.25, m	2′	78.1	3.37, d (8.3)
4′	71.5	3.29, d (8.6)	3′	79.7	3.45, m
5′	78.1	3.33, d (8.7)	4′	72.2	3.20, t (9.2)
6′	62.7	3.85, d (8.5) 3.69, dd (11.9, 5.4)	5′	77.8	3.16, m
No.	**31**		6′	63.0	3.81, dd (11.5, 2.3) 3.59, dd (11.5, 5.6)
	*δ*_C_	*δ*_H_	1″	101.6	5.35, s
1	91.3	4.70, s	2″	72.2	3.90, dd (3.5, 1.7)
2	99.3	-	3″	72.3	3.69, dd (9.5, 3.4)
3	39.1	2.54, dd (14.0, 4.7) 2.10, dd (13.9, 6.4)	4″	74.1	3.40, m
4	72.5	4.27, m	5″	69.8	4.03, dq (9.6, 6.2)
5	87.3	3.80, q (4.3)	6″	18.1	1.30, d (6.2)
6	63.6	3.61, m			
7	163.6	-			
1′	68.6	3.58, m 3.49, m			
2′	32.9	1.58, m			
3′	20.4	1.42, m			
4′	14.2	0.94, t			

**Table 3:** Anti-HepG2 cell proliferation activity of compounds **1**, **8**, **10**, and **29**–**31**.

Compound	IC_50_	
	24 h	48 h
PTX *	0.52 ± 0.10	0.46 ± 0.04
**1**	20.15 ± 0.04	/
**8**	27.91 ± 0.65	19.73 ± 0.35
**10**	30.47 ± 21.55	17.57 ± 0.10
**29**	16.07 ± 0.22	19.83 ± 0.59
**30**	/	19.8 ± 0.63
**31**	20.05 ± 0.50	/

Data are represented as the mean value ± SD, n = 2. * PTX was employed as the positive control.

## Data Availability

The original contributions presented in this study are included in the article/supplementary material. Further inquiries can be directed to the corresponding author(s).

## Supplementary Materials

The following supporting information can be downloaded at: https://www.mdpi.com/article/10.3390/ijms262211216/s1.
