# Supplementary material for: Chemical Constituents and Antiproliferative Activity Against RAFLs and HepG2 Cells of Clematis henryi
Source: Int J Mol Sci. 2025 Nov 20;26(22):11216. doi: 10.3390/ijms262211216 (PMC12653648; doi:10.3390/ijms262211216)
Supplement: Supplementary file 1 [file ijms-26-11216-s001.zip › ijms-3959456-supplementary.pdf]

Supplementary Materials

Chemical Constituents and Antiproliferative Activity Against RAFLs and HepG2 Cells of Clematis henryi

Contents:

Figure S1. ^{1}H NMR spectrum of henriside A (**1**) (CD_3OD, 600 MHz).
Figure S2. ^{13}C NMR spectrum of henriside A (**1**) (CD_3OD, 151 MHz).
Figure S3. DEPT 135° spectrum of henriside A (**1**) (CD_3OD, 151 MHz).
Figure S4. HSQC spectrum of henriside A (**1**).
Figure S5. HMBC spectrum of henriside A (**1**).
Figure S6. ^{1}H-^{1}H COSY spectrum of henriside A (**1**).
Figure S7. ^{1}H NMR spectrum of henriside B (**29**) (CD_3OD, 600 MHz).
Figure S8. ^{13}C NMR spectrum of henriside B (**29**) (CD_3OD, 151 MHz).
Figure S9. DEPT 135° spectrum of henriside B (**29**) (**1**) (CD_3OD, 151 MHz).
Figure S10. HSQC spectrum of henriside B (**29**).
Figure S11. HMBC spectrum of henriside B (**29**).
Figure S12. ^{1}H-^{1}H COSY spectrum of henriside B (**29**).
Figure S13. ^{1}H NMR spectrum of henriside C (**30**) (CD_3OD, 600 MHz).
Figure S14. ^{13}C NMR spectrum of henriside C (**30**) (CD_3OD, 151 MHz).
Figure S15. DEPT 135° spectrum of henriside C (**30**) (**1**) (CD_3OD, 151 MHz).
Figure S16. HSQC spectrum of henriside C (**30**).
Figure S17. HMBC spectrum of henriside C (**30**).
Figure S18. ^{1}H-^{1}H COSY spectrum of henriside C (**30**).
Figure S19. ^{1}H NMR spectrum of henriside D (**31**) (CD_3OD, 600 MHz).
Figure S20. ^{13}C NMR spectrum of henriside D (**31**) (CD_3OD, 151 MHz).
Figure S21. DEPT 135° spectrum of henriside D (**31**) (CD_3OD, 151 MHz).
Figure S22. HSQC spectrum of henriside D (**31**).
Figure S23. HMBC spectrum of henriside D (**31**).
Figure S24. ^{1}H-^{1}H COSY spectrum of henriside D (**31**).

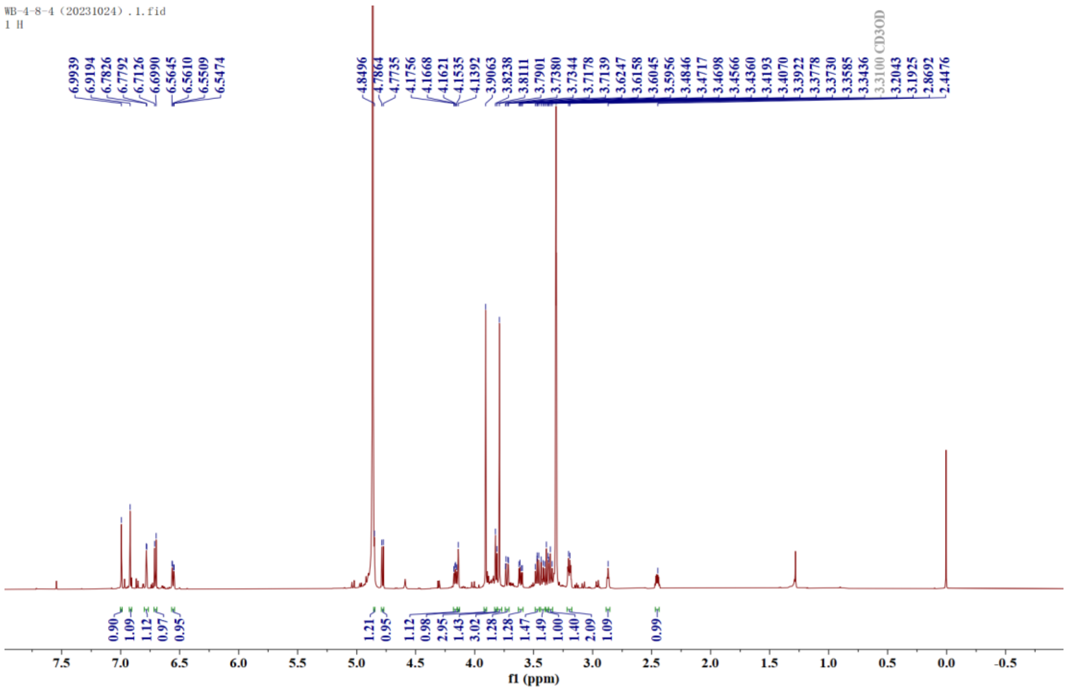

Figure S1. ^{1}H NMR spectrum of henriside A (**1**) (CD_3OD, 600 MHz)

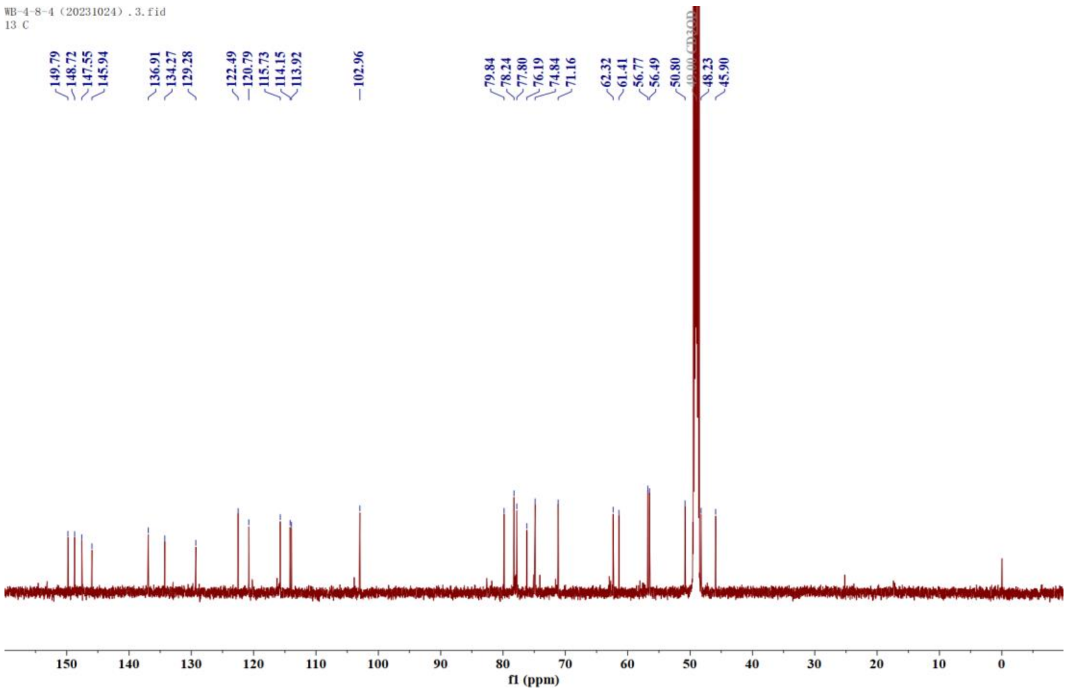

Figure S2. ^{13}C NMR spectrum of henriside A (**1**) (CD_3OD, 151 MHz)

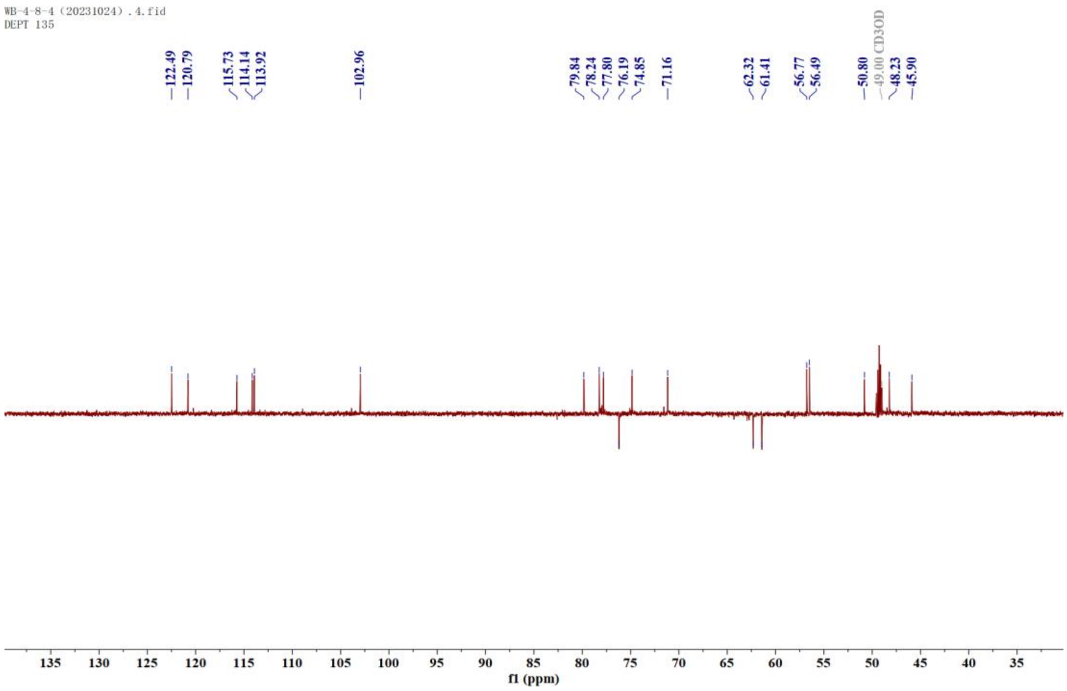

Figure S3. DEPT 135° spectrum of henriside A (**1**) (CD_3OD, 151 MHz)

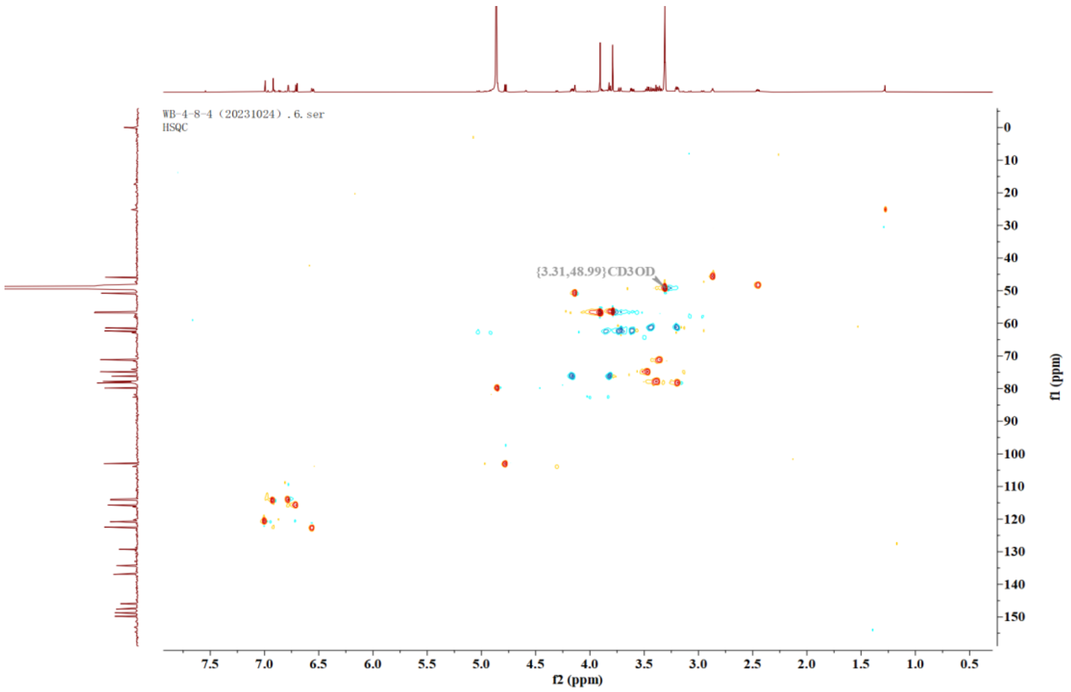

Figure S4. HSQC spectrum of henriside A (**1**)

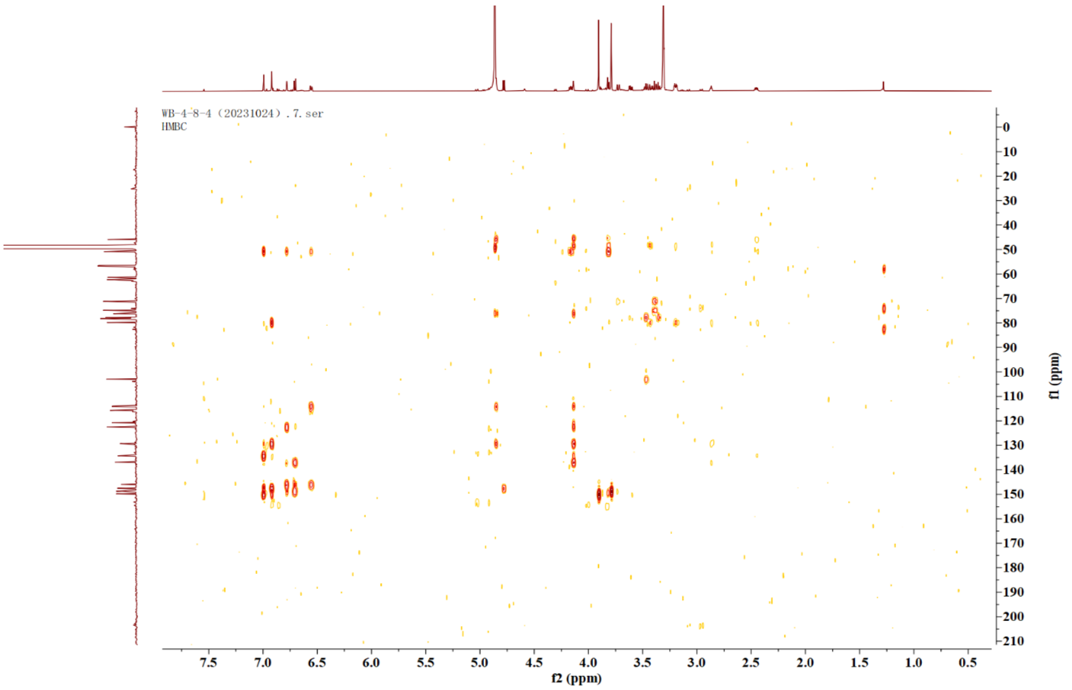

Figure S5. HMBC spectrum of henriside A (**1**)

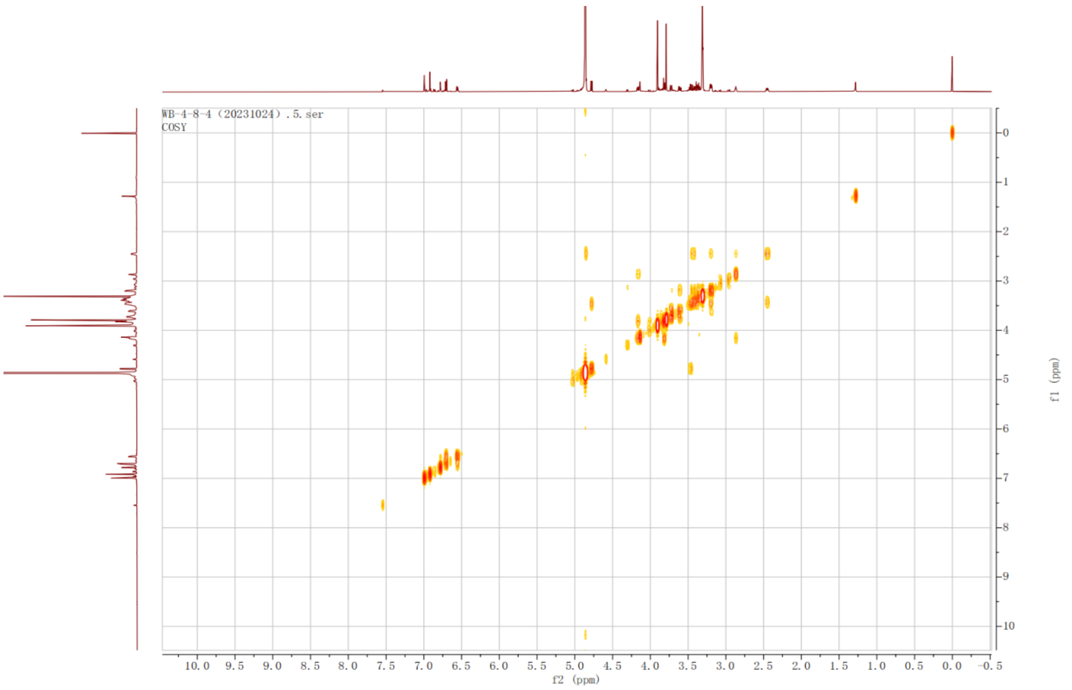

Figure S6. ^{1}H-^{1}H COSY spectrum of henriside A (**1**)

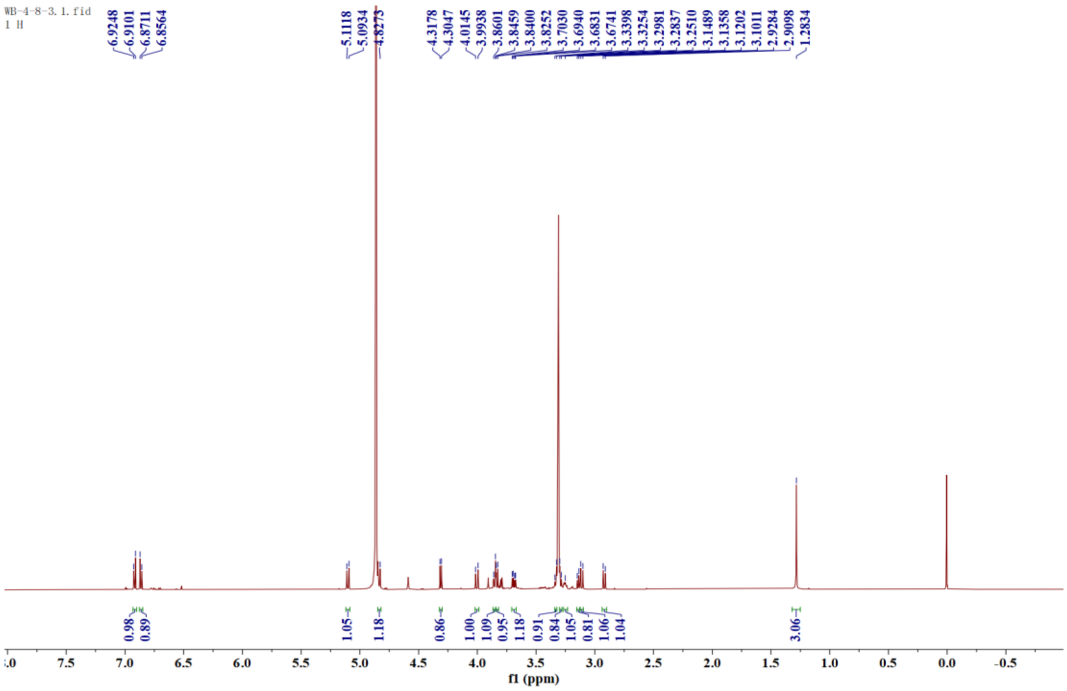

Figure S7. ^{1}H NMR spectrum of henriside B (**29**) (CD_3OD, 600 MHz)

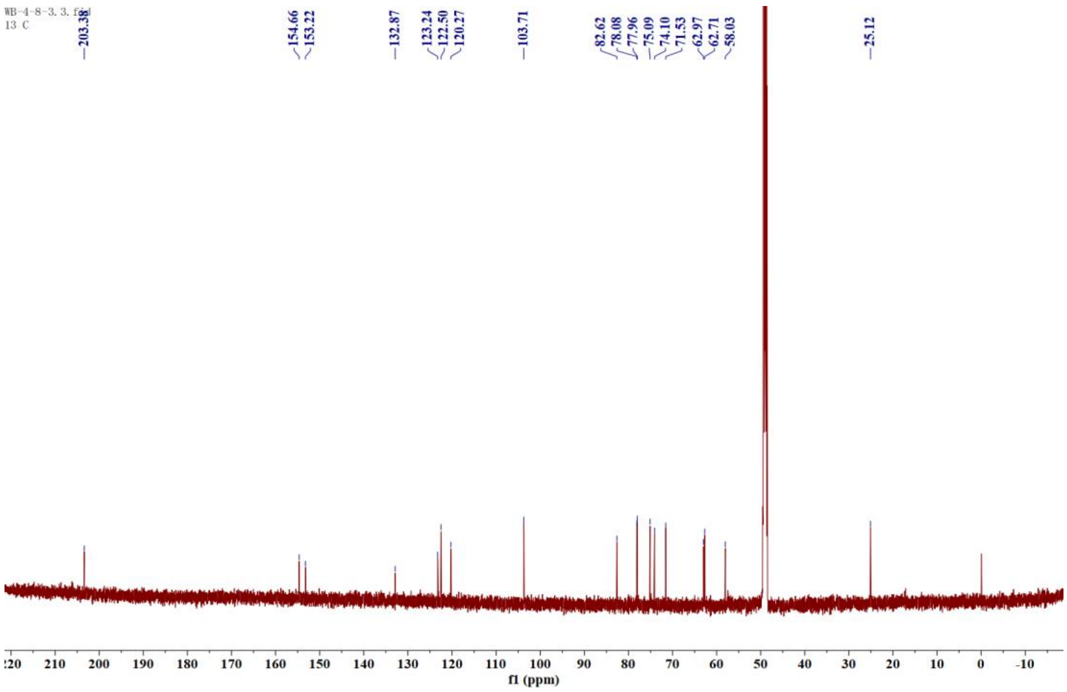

Figure S8. ^{13}C NMR spectrum of henriside B (**29**) (CD_3OD, 151 MHz)

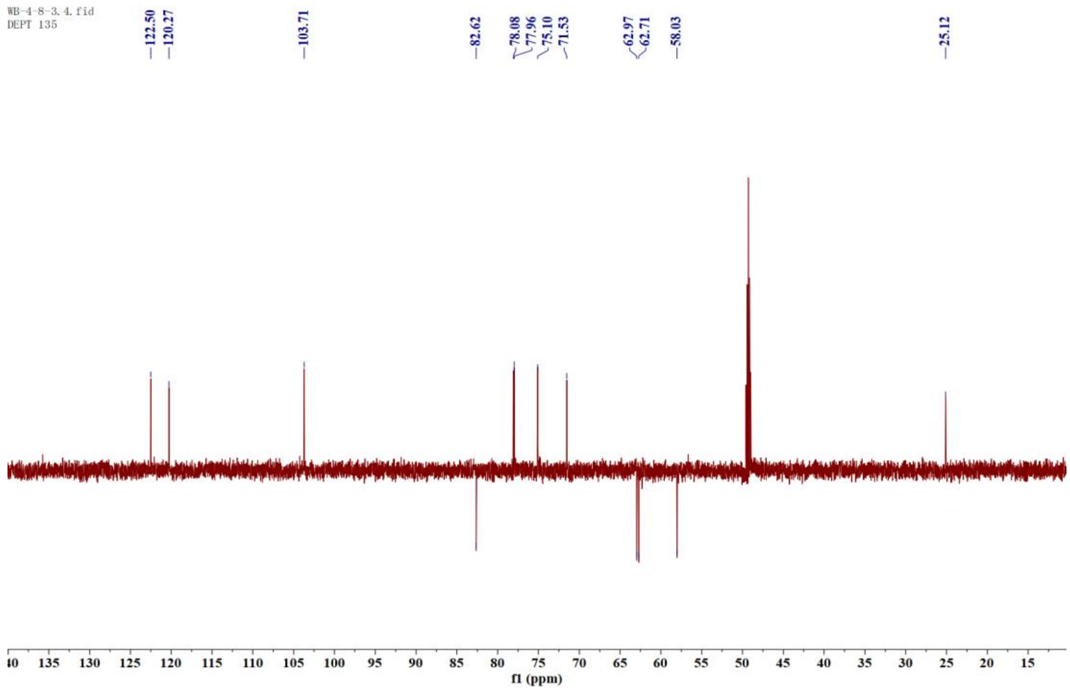

Figure S9. DEPT 135° spectrum of henriside B (**29**) (CD_3OD, 151 MHz)

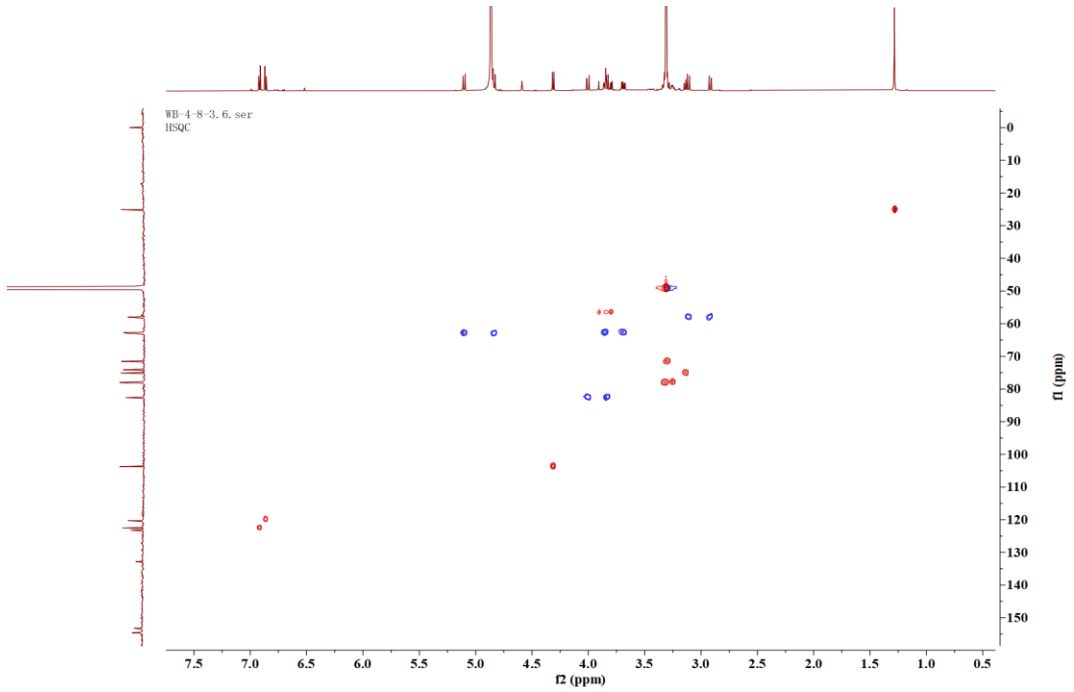

Figure S10. HSQC spectrum of henriside B (**29**)

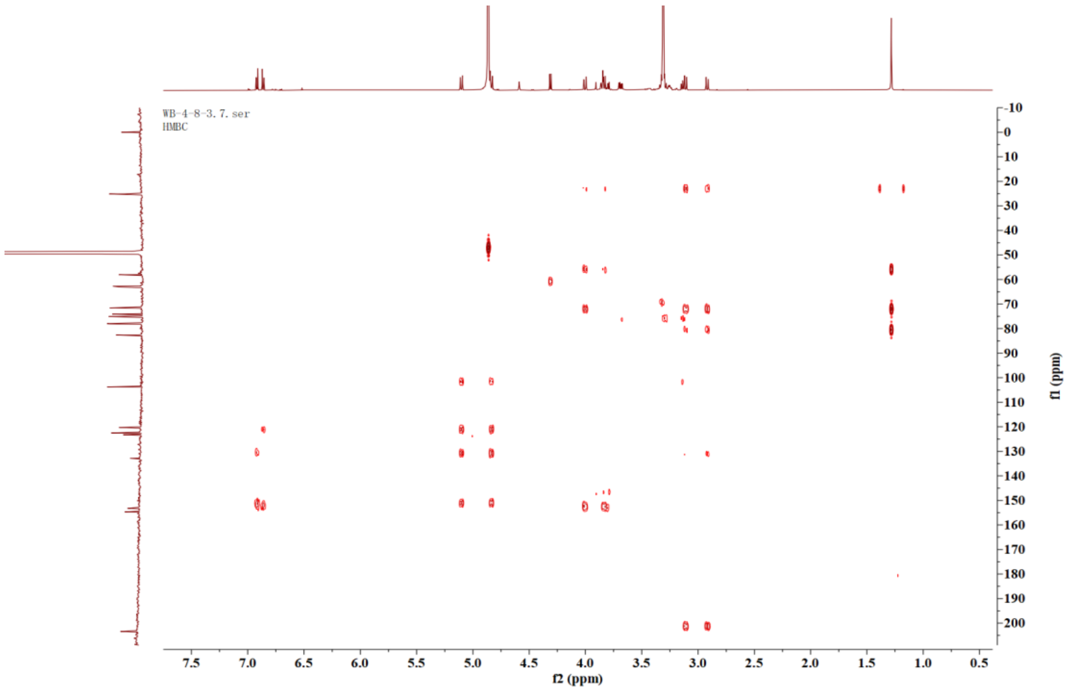

Figure S11. HMBC spectrum of henriside B (**29**)

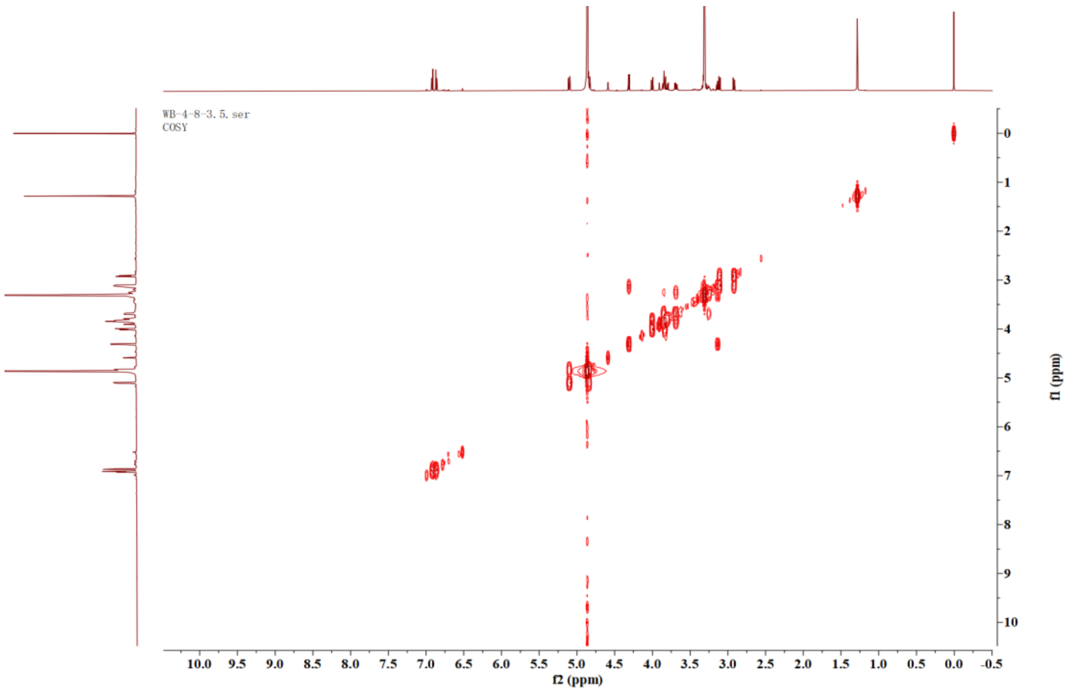

Figure S12. ^{1}H-^{1}H COSY spectrum of henriside B (**29**)

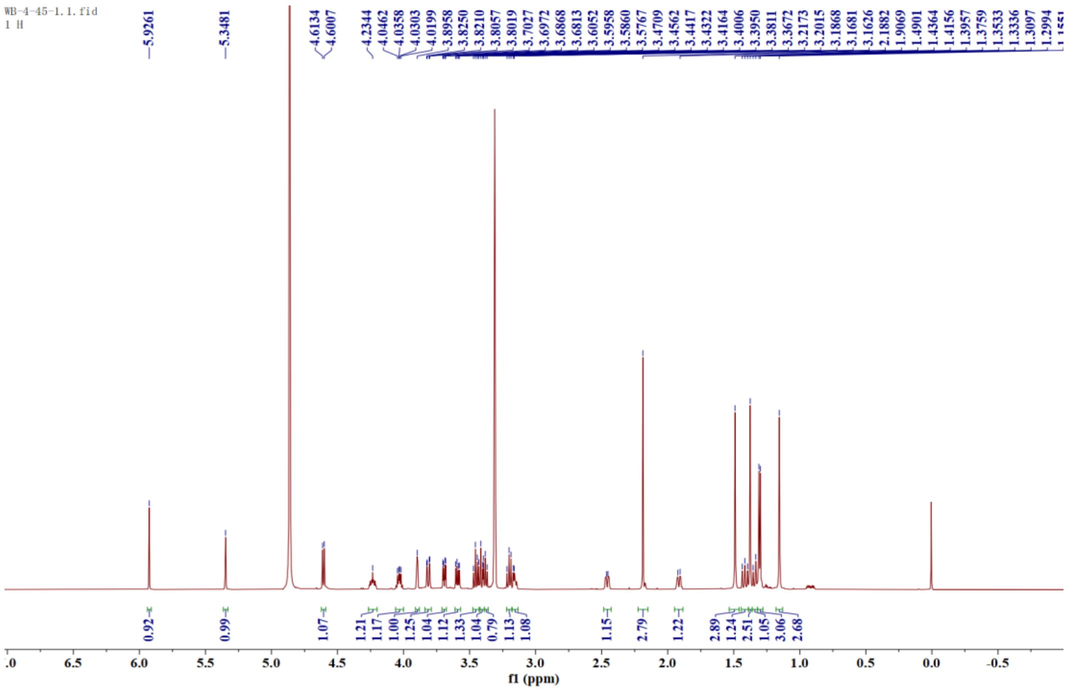

Figure S13. ^{1}H NMR spectrum of henriside C (**30**) (CD_3OD, 600 MHz)

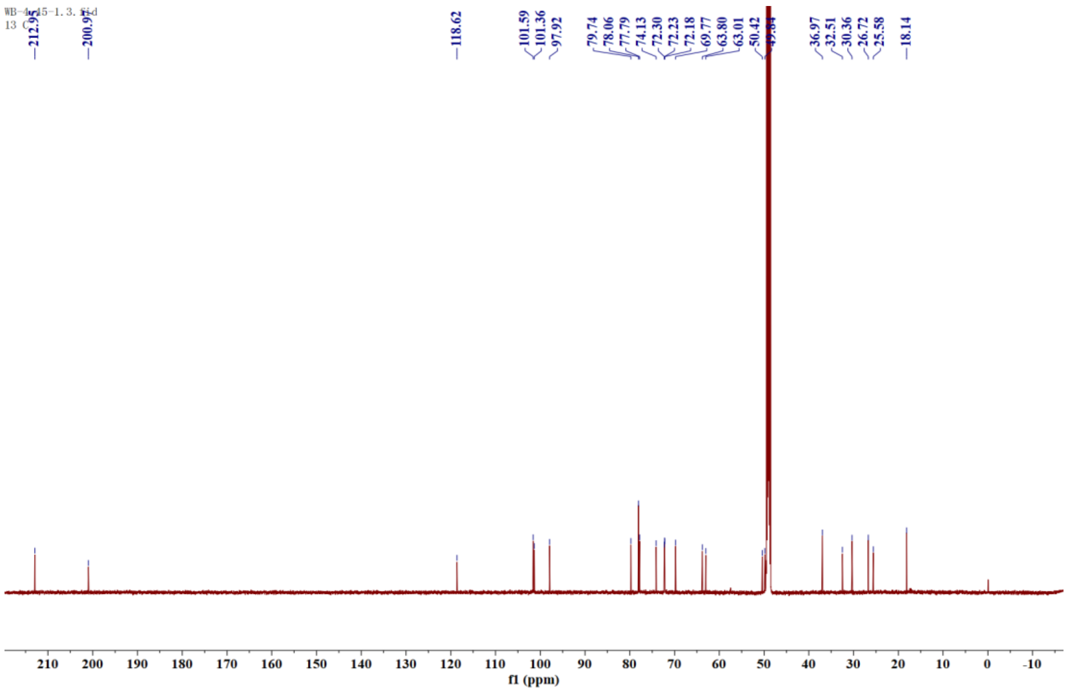

Figure S14. ^{13}C NMR spectrum of henriside C (**30**) (CD_3OD, 151 MHz)

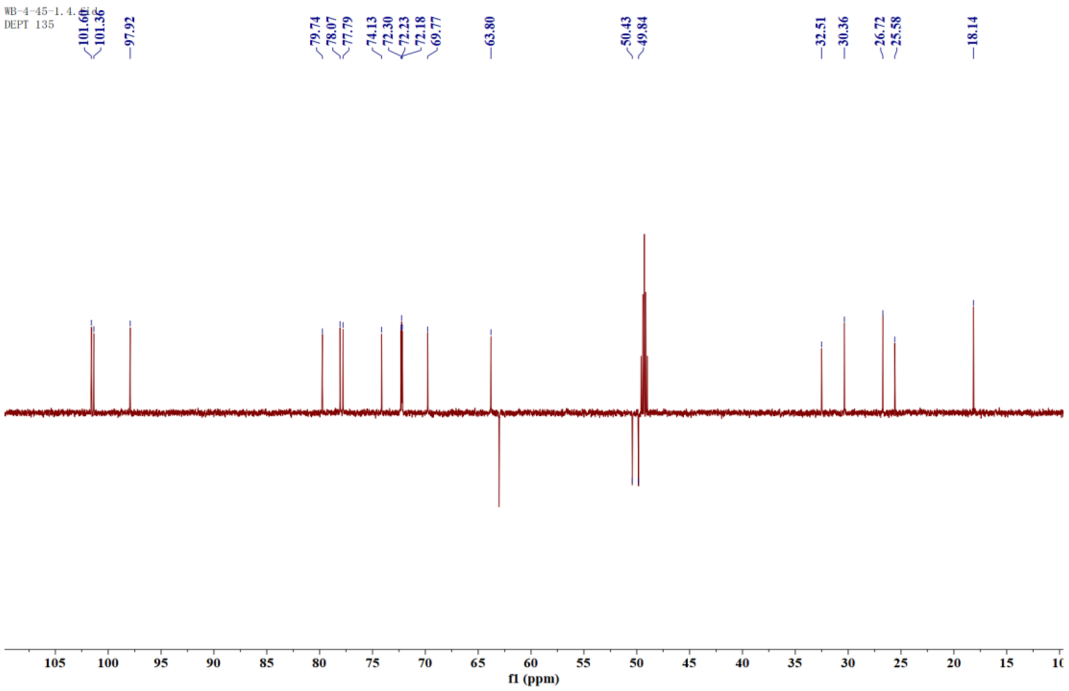

Figure S15. DEPT 135° spectrum of henriside C (**30**) (CD_3OD, 151 MHz)

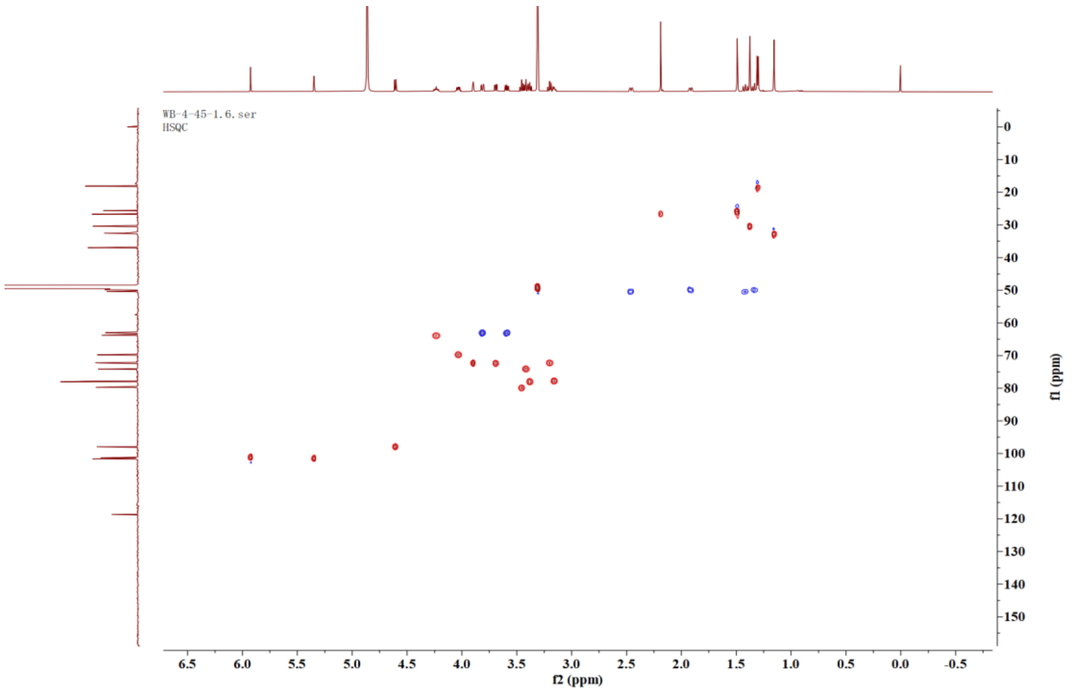

Figure S16. HSQC spectrum of henriside C (**30**)

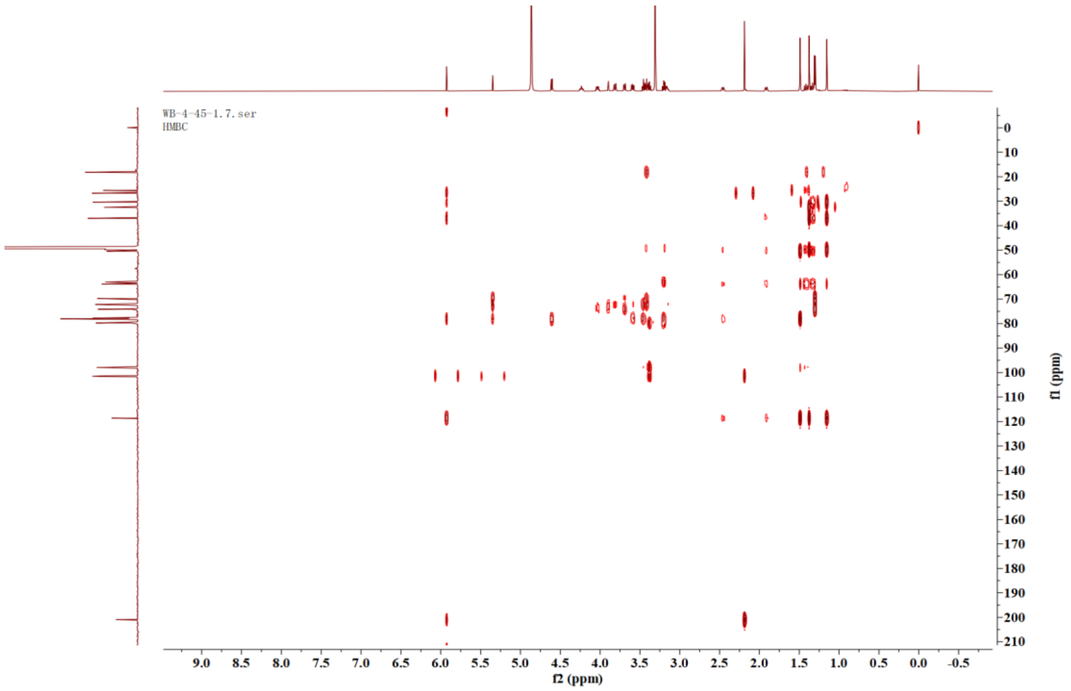

Figure S17. HMBC spectrum of henriside C (**30**)

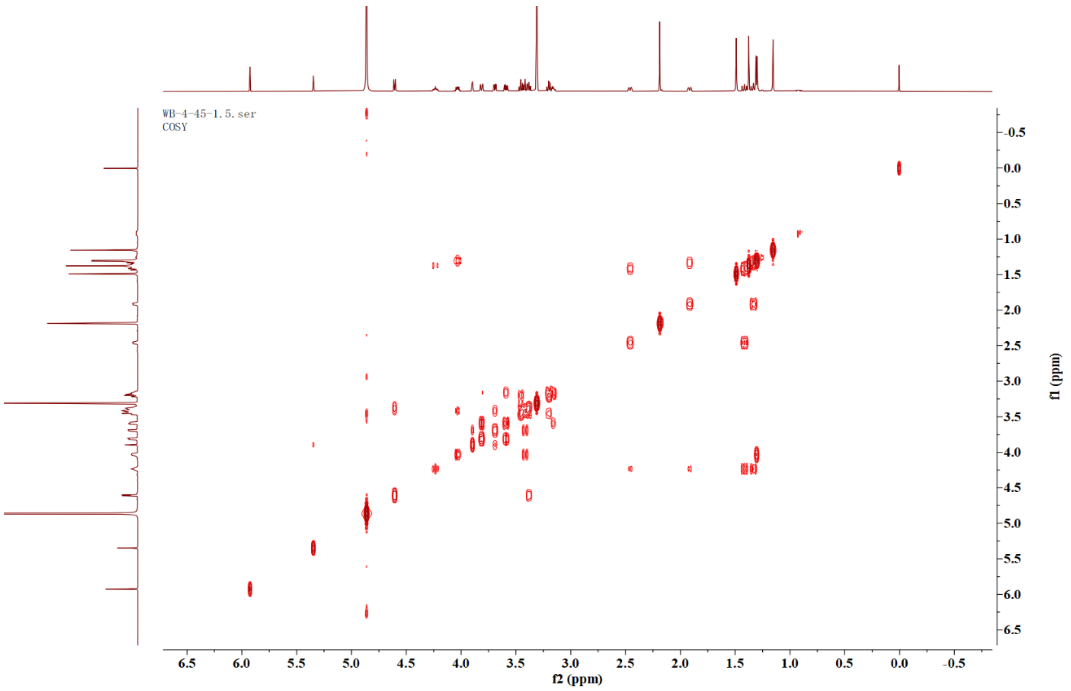

Figure S18. ^{1}H-^{1}H COSY spectrum of henriside C (**30**)

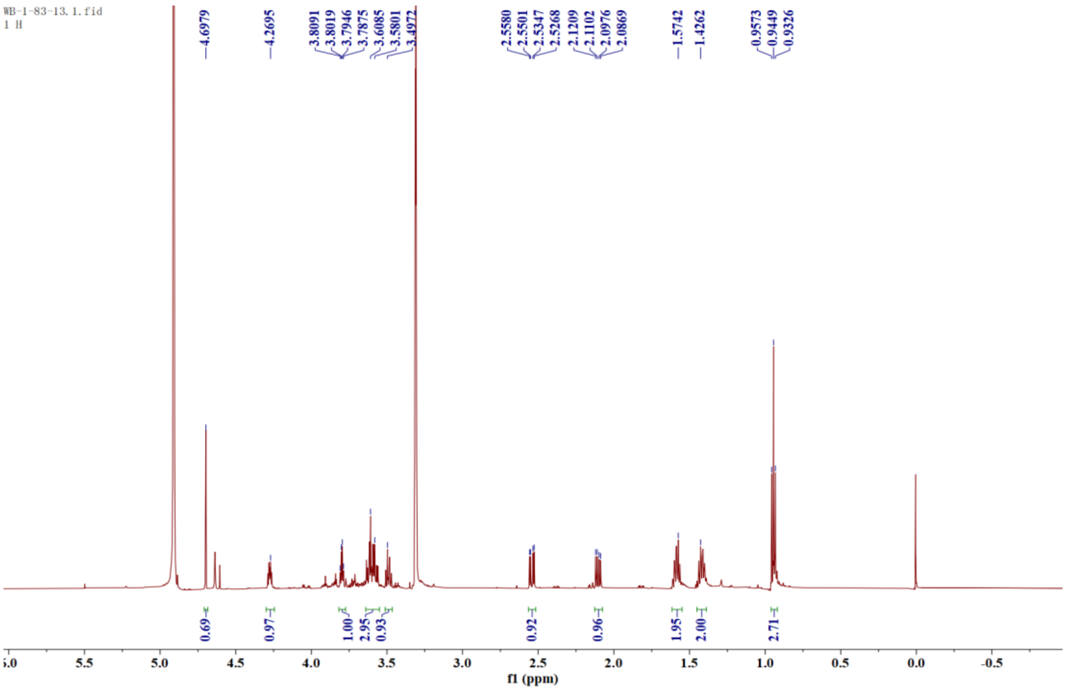

Figure S19. ^{1}H NMR spectrum of henriside D (**31**) (CD_3OD, 600 MHz)

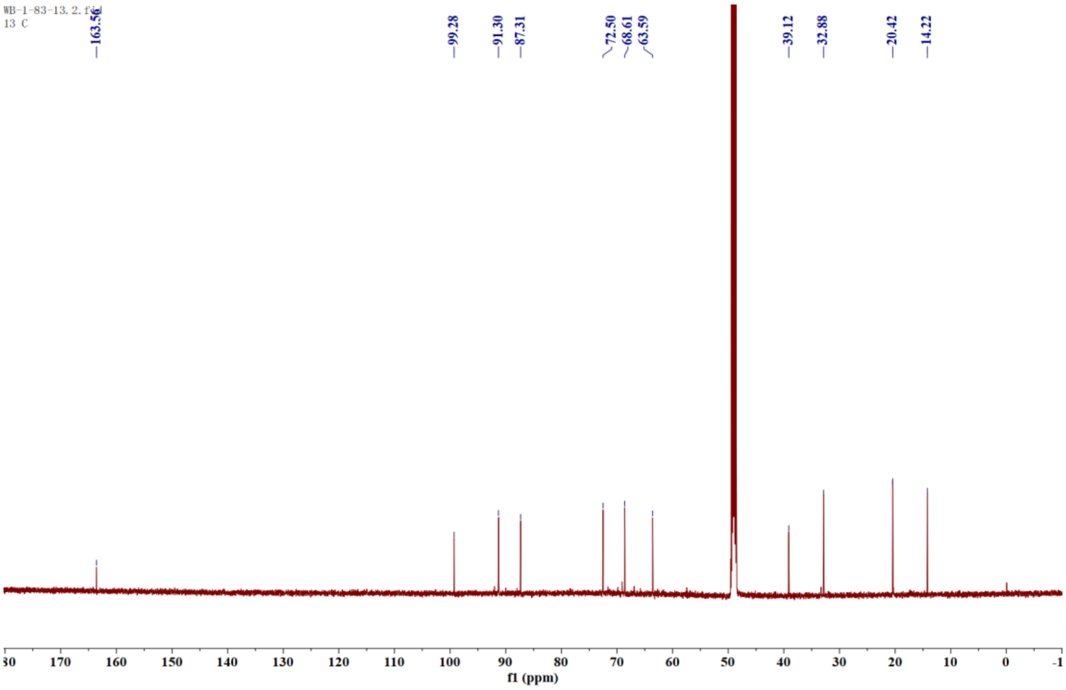

Figure S20. ^{13}C NMR spectrum of henriside D (**31**) (CD_3OD, 151 MHz)

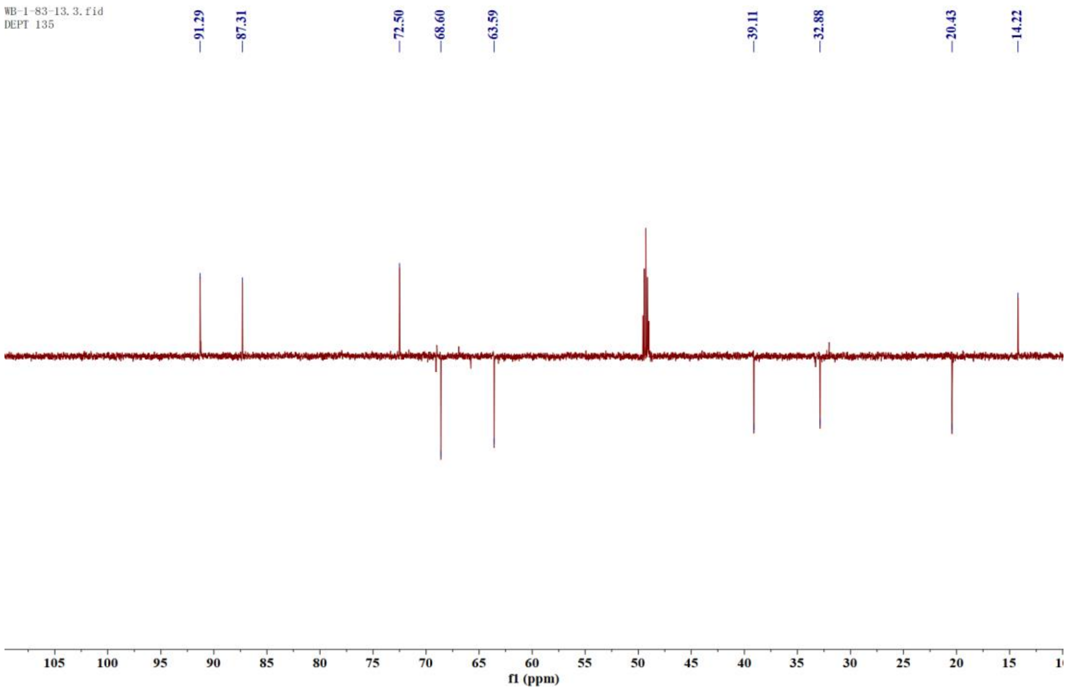

Figure S21. DEPT 135° spectrum of henriside D (**31**) (CD_3OD, 151 MHz)

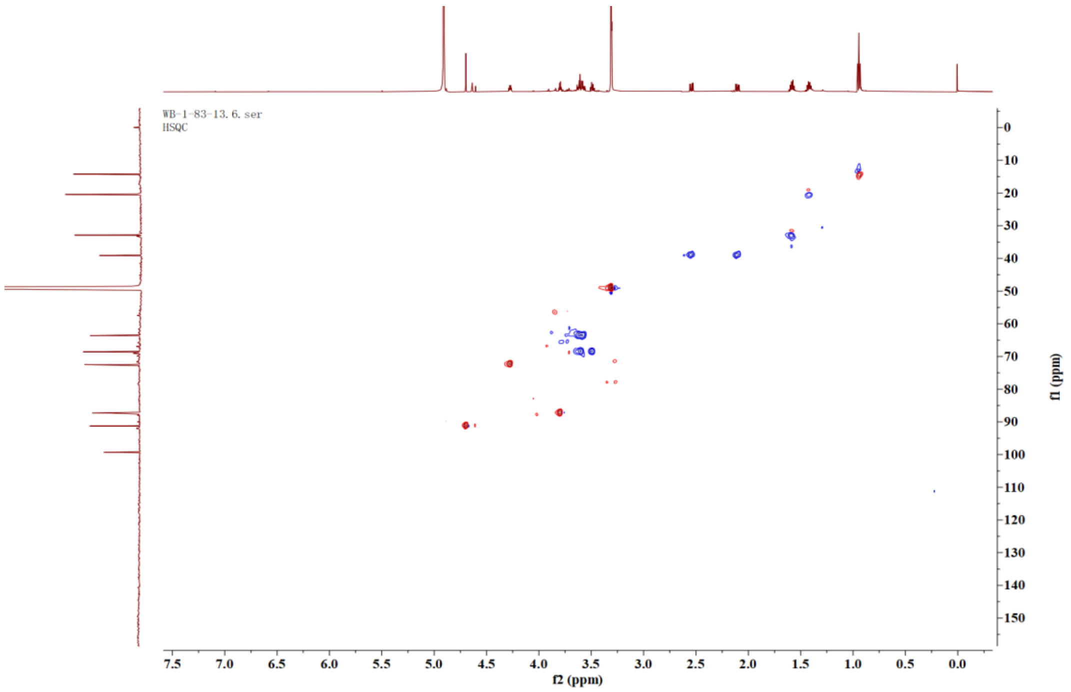

Figure S22. HSQC spectrum of henriside D (**31**)

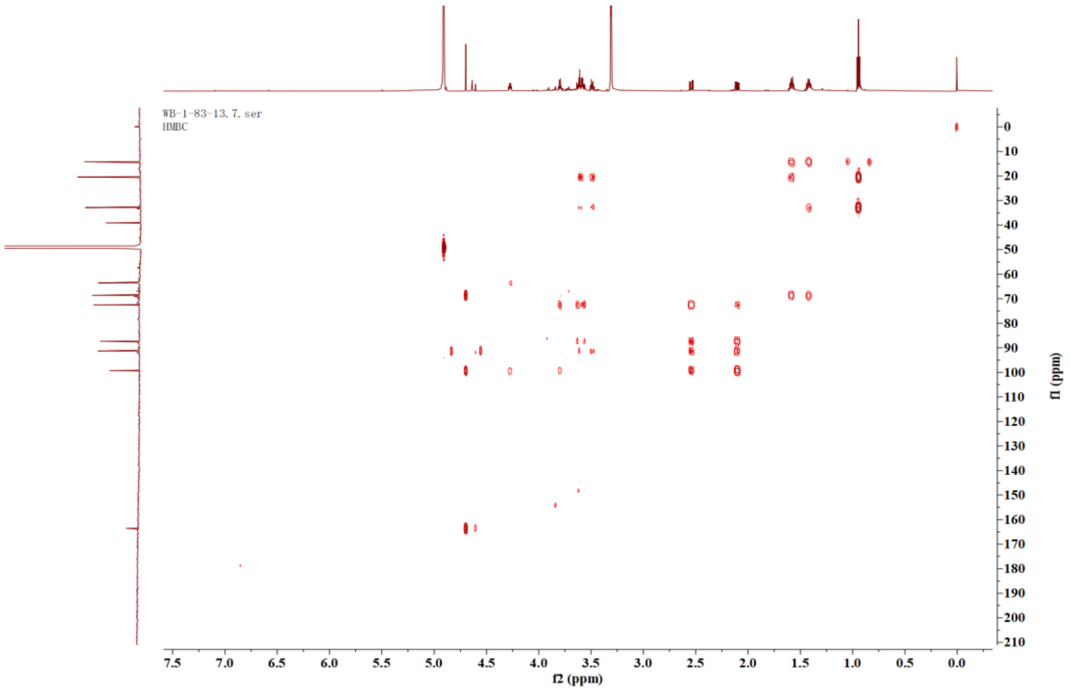

Figure S23. HMBC spectrum of henriside D (**31**)

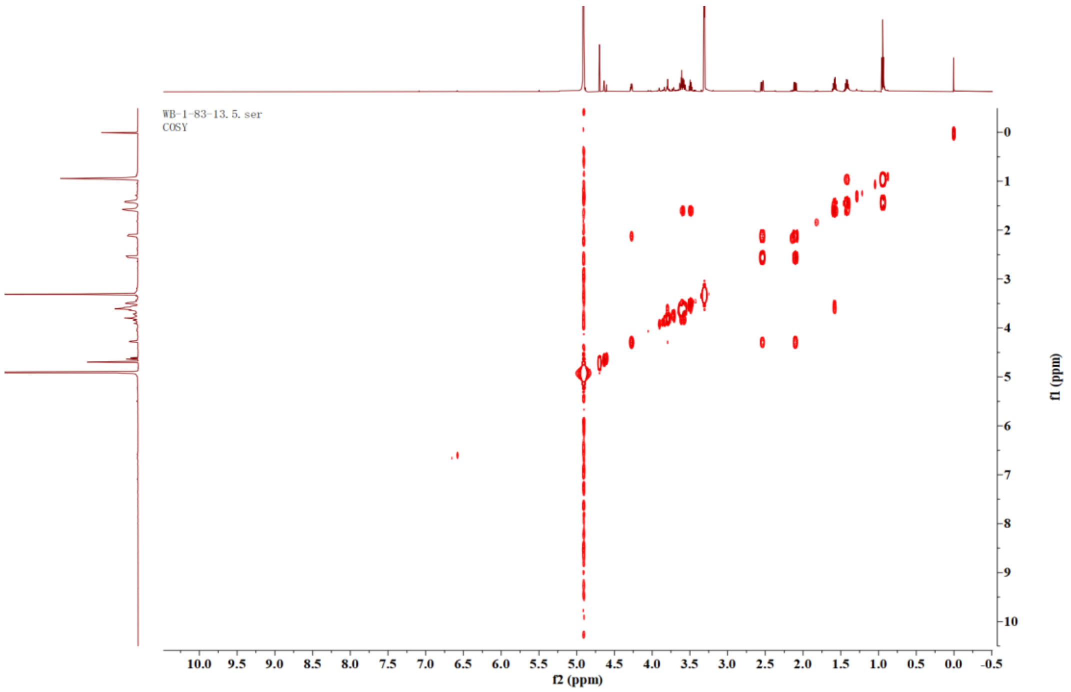

Figure S24. ^{1}H-^{1}H COSY spectrum of henriside D (**31**)
